# O antigen biogenesis sensitises *Escherichia coli* K-12 to bile salts, providing a plausible explanation for its evolutionary loss

**DOI:** 10.1371/journal.pgen.1010996

**Published:** 2023-10-04

**Authors:** Jilong Qin, Yaoqin Hong, Renato Morona, Makrina Totsika

**Affiliations:** 1 Centre for Immunology and Infection Control, School of Biomedical Sciences, Queensland University of Technology, Brisbane, Queensland, Australia; 2 Max Planck Queensland Centre, Queensland University of Technology, Brisbane, Queensland, Australia; 3 School of Biological Sciences, Department of Molecular & Biomedical Sciences, Research Centre for Infectious Diseases, University of Adelaide, Adelaide, Australia; Uppsala University, SWEDEN

## Abstract

*Escherichia coli* K-12 is a model organism for bacteriology and has served as a workhorse for molecular biology and biochemistry for over a century since its first isolation in 1922. However, *Escherichia coli* K-12 strains are phenotypically devoid of an O antigen (OAg) since early reports in the scientific literature. Recent studies have reported the presence of independent mutations that abolish OAg repeating-unit (RU) biogenesis in *E*. *coli* K-12 strains from the same original source, suggesting unknown evolutionary forces have selected for inactivation of OAg biogenesis during the early propagation of K-12. Here, we show for the first time that restoration of OAg in *E*. *coli* K-12 strain MG1655 synergistically sensitises bacteria to vancomycin with bile salts (VBS). Suppressor mutants surviving lethal doses of VBS primarily contained disruptions in OAg biogenesis. We present data supporting a model where the transient presence and accumulation of lipid-linked OAg intermediates in the periplasmic leaflet of the inner membrane interfere with peptidoglycan sacculus biosynthesis, causing growth defects that are synergistically enhanced by bile salts. Lastly, we demonstrate that continuous bile salt exposure of OAg-producing MG1655 in the laboratory, can recreate a scenario where OAg disruption is selected for as an evolutionary fitness benefit. Our work thus provides a plausible explanation for the long-held mystery of the selective pressure that may have led to the loss of OAg biogenesis in *E*. *coli* K-12; this opens new avenues for exploring long-standing questions on the intricate network coordinating the synthesis of different cell envelope components in Gram-negative bacteria.

## Author summary

*Escherichia coli* K-12 is the most studied microorganism, widely used in laboratories for studying bacteriology and as a tool for molecular biology. *E*. *coli* K-12 strains originating from the same institute harbour independent inactivation of O antigen biosynthesis and assembly pathways, strongly suggesting that *E*. *coli* K-12 was originally producing an O antigen. However, the reason why it is devoid of O antigen, where most other *E*. *coli* lineages are not, remains a long-standing question. Our work has uncovered a previously unknown selection pressure of bile salts on bacterial O antigen biogenesis, which provides a plausible scenario for how the early propagation of *E*. *coli* K-12 strains in bile salt-containing media could have led to loss of O antigen in K-12. Our results also suggest that the accumulation of O antigen intermediates in the bacterial periplasm may interfere with bacterial cell wall synthesis, which opens a new avenue of research into the interplay of different cell envelope component synthesis pathways.

## Introduction

**Fig 1:**
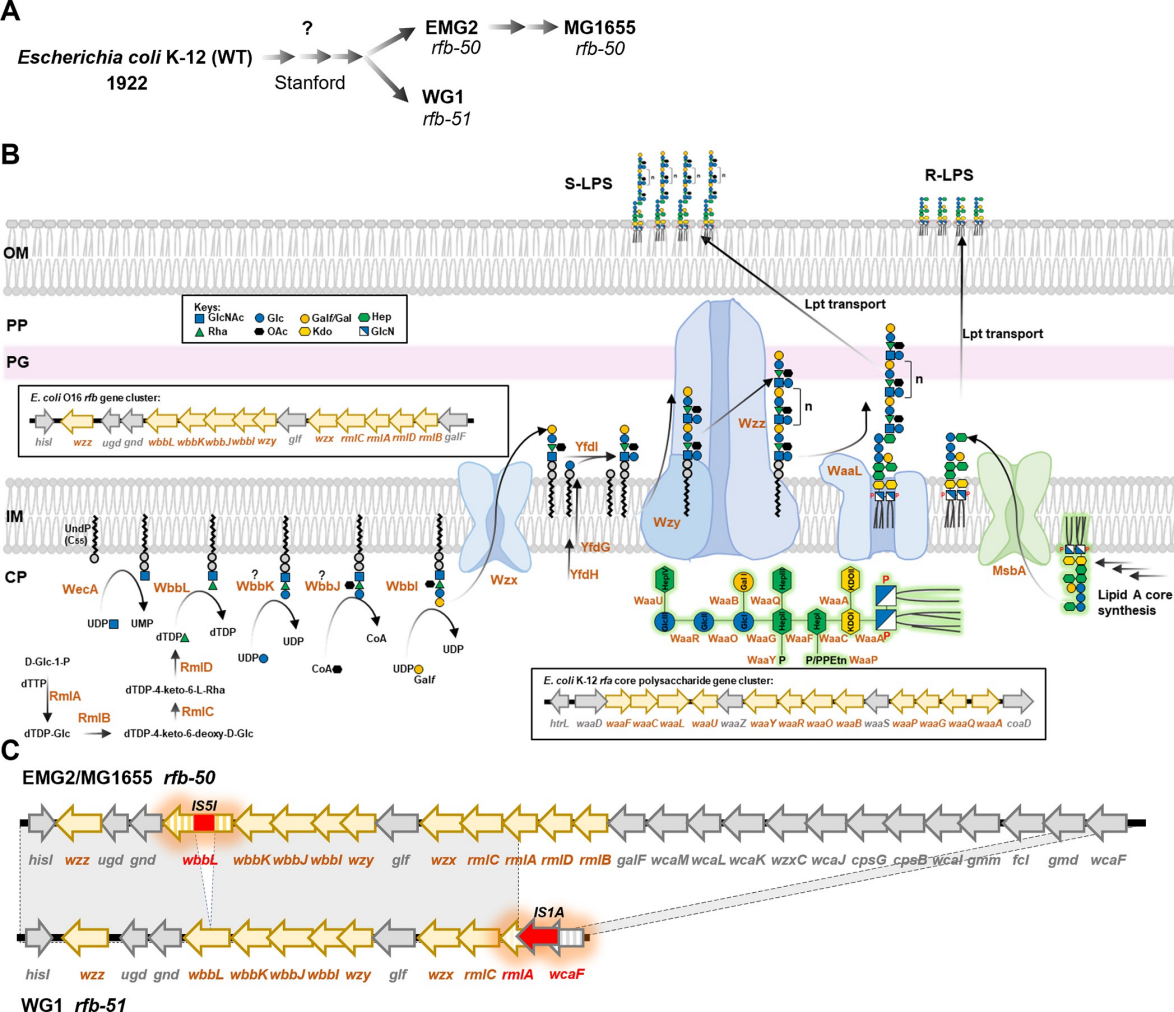
The biogenesis of O16-LPS in *E*. *coli* K-12. (A) Schematic representation of the early history of *E*. *coli* K-12 wild-type strains and derivatives, denoting relevant *rfb* mutations. Details of the procedures and media used during isolation and early propagation of strains are unknown (marked by question mark) but can be inferred by common practices at the time. (B) Biogenesis of O16-LPS in the original WT *E*. *coli* K-12 strain. The enzymatic order of glycosyltransferases WbbK and WbbJ is marked with question marks as it remains to be experimentally determined and the order presented is predicted from the inverted gene order within the operon. The structure of Lipid A-core is shown in green glow with corresponding glycosyltransferases and kinases. Genes that are responsible for the biosynthesis of C_55_-PP-OAg^O16^ and K-12 lipid A-core are shown in box. S-LPS, smooth LPS; R-LPS rough LPS. OM, outer membrane; PP periplasm; PG, peptidoglycan; IM, inner membrane; CP, cytoplasm, n refers to the modal chain length of polymerised OAg RU in MG1655-S. (C) Schematic representation of sequence alignment between regions *rfb-50* from EMG2/MG1655 and *rfb-51* from WG1. Grey shading marks areas of 100% sequence identity.

*Escherichia coli* K-12, the most intensively studied microorganism, has served as a model organism for modern bacteriology and as a workhorse for biochemistry, genetics, and molecular biology. The wild-type strain of *E*. *coli* K-12 was first isolated in 1922 from the stool of a diphtheria patient and was soon after deposited in the strain collection of the Department of Bacteriology at Stanford University, where it was used in the teaching laboratories and maintained in stab cultures (Fig 1A) [1,2]. The first report of *E*. *coli* K-12 in the scientific literature was in 1944 [3] where it was acquired from Dr. C. E. Clifton (Dept. of Bacteriology, Stanford University). This culture was taken to Europe and shared with W. Hayes where it was given the name EMG2 [4]. The EMG2 strain subsequently gave rise to strain MG1655 [5], which has since been used worldwide as a wild-type *E*. *coli* K-12 strain, the chromosome of which was the source for the first fully-sequenced *E*. *coli* genome published in 1997 [6] (Fig 1A). The original *E*. *coli* K-12 culture shared from the Tatum laboratory was lost in the Lederberg laboratory and was replaced by another subculture of K-12 sourced from the collection of the Department of Bacteriology at Stanford University that was given the name WG1 (Fig 1A) [2]. After 30 years of maintenance in laboratories, all *E*. *coli* K-12 strains were reported by serological studies to be devoid of antigenic structures (O and K antigens) typically found on newly isolated wild-type strains [7] and were also shown to fail to colonise the human gut [8].

The O antigen (OAg) is a major component of bacterial surface lipopolysaccharide (LPS) molecules and in *E*. *coli* is responsible both for serological specificity (due to its high antigenic variability [9]) and for host gut colonisation [10]. Presentation of OAg-capped LPS (termed smooth LPS, S-LPS) on the bacterial cell surface requires synthesis of complete core oligosaccharides structure (by the *rfa* gene cluster) on lipid A, as well as synthesis of OAg repeating units (RUs) (by the *rfb* gene cluster). *E*. *coli* K-12 was shown to synthesise the complete core oligosaccharides structure [11] (termed rough LPS, R-LPS) using glycosyltransferases and kinases encoded by *waa* genes in the *rfa* region (Fig 1B), and was able to be substituted with other OAg when carrying cloned *rfb* gene clusters from other strains [12,13], suggesting that *E*. *coli* K-12 is defective in its chromosomal *rfb* region. The K-12 *rfb* region contains genes encoding dTDP-L-rhamnose synthesis enzymes (RmlABCD) and glycosyltransferases for O16 OAg (OAg^O16^) [14,15] (Fig 1B). OAg^O16^ is synthesised on the universal lipid carrier undecaprenol phosphate (C_55_-P), on which the addition of monosaccharides and chemical modifications is sequentially catalysed by WecA, WbbL, WbbK, WbbJ and WbbI to produce the complete O16 repeat unit (RU^O16^) in the cytosolic face of the inner membrane (IM) [16] (Fig 1B). The complete unit of C_55_-PP-RU^O16^ is then flipped across the IM by the Wzx flippase to the periplasmic face of the IM and modified by glucosyltransferase YfdI. RUs are then either polymerised by Wzy (to modal lengths, denoted as n in Fig 1B, specified by the co-polymerase Wzz), or transferred to lipid A-core oligosaccharide molecules by WaaL in a competing manner [17].

K-12 strains EMG2 and WG1 (both sourced from Stanford University) were found to carry mutations in the OAg gene cluster (*rfb*) [14] that led to production of R-LPS in both strains (Fig 1B). Despite their common phenotype, independent mutations were mapped in *rfb*, with EMG2 carrying the *rfb-50* mutation and WG1 carrying the *rfb-51* mutation [14]. The *rfb-50* mutation in strain EMG2 is due to an IS5I insertion element in the coding sequence of WbbL, the second glycosyltransferase of RU^O16^, abolishing the production of OAg^O16^. Mutation *rfb-51* in strain WG1 involves a large deletion between the dTDP-L-rhamnose synthesis gene *rmlA* and the colanic acid synthesis gene *wcaF* (Fig 1C) [18]. A plasmid carrying the intact *wbbL* from WG1 restored production of S-LPS in the EMG2 strain [14], confirming the independency of *rfb* mutational events in these two K-12 strains originating from the same institute. This evidence strongly suggests that *E*. *coli* K-12 was once producing an OAg (i.e. had S-LPS). However, all K-12 strains appear to lack OAg since their first report in literature [3], suggesting that loss of OAg occurred early in the original isolate’s history, possibly during strain maintenance at Stanford University from the 1920s to 1940s. It is likely that certain culture conditions used at the time for the early propagation of *E*. *coli* K-12 strains at Stanford University were unfavourable to OAg production, and led to evolutionary selection of two independent *rfb* mutations in strains WG1 and EMG2.

Isolation and identification of *E*. *coli* strains in the early 20^th^ century relied on specialised culture media, including MacConkey medium [19], which through the presence of bile salts and crystal violet offered some selectivity by inhibiting the growth of Gram-positive bacteria [20]. MacConkey medium has since been used widely among bacteriologists, teachers and practitioners since 1922 [21], as well as in clinical laboratories to identify bacteria from patient stool samples [22,23]. Through experimentation, we herein explored the selective stress that MacConkey medium could have posed to *E*. *coli* K-12 for loss of its OAg production. We report that restoration of OAg production in *E*. *coli* K-12 sensitised bacteria towards vancomycin when grown on MacConkey agar due to the presence of bile salt (BS). We experimentally demonstrate that combining vancomycin with bile salts (VBS) selects for loss of OAg in *E*. *coli* K-12 strains genetically restored to produce smooth O16 LPS, and that the presence of the C_55_-PP-OAg intermediates in the periplasm during production of O16 OAg is what sensitised bacteria to BS. Lastly, we demonstrate that culturing smooth *E*. *coli* K-12 in media supplemented with BS rapidly selects for R-LPS mutants. Taken together our data provide the most plausible explanation to the long-standing mystery of how *E*. *coli* K-12 lost its OAg during early strain maintenance in the laboratory and shed new light on the interplay between different cell envelope component synthesis pathways in Gram-negative bacteria.

## Results

### Restoration of OAg production sensitises *E*. *coli* K-12 to vancomycin in the presence of bile salts

To investigate the cause of the loss of OAg in *E*. *coli* K-12, we firstly restored production of OAg^O16^ in the K-12 reference strain MG1655. An isogenic MG1655-S (‘-S’ for smooth) variant was engineered by flawless allelic exchange with an intact *wbbL* PCR amplicon (S1A and S1B Fig) followed by selection with Colicin E2, a DNase that is unable to enter bacterial cells when its outer membrane receptor BtuB is masked by OAg [24]. Strain MG1655-S was genotypically confirmed by whole genome sequencing and was phenotypically confirmed to produce OAg^O16^ that conferred resistance to Colicin E2 (S1C–S1E Fig).

**Fig 2:**
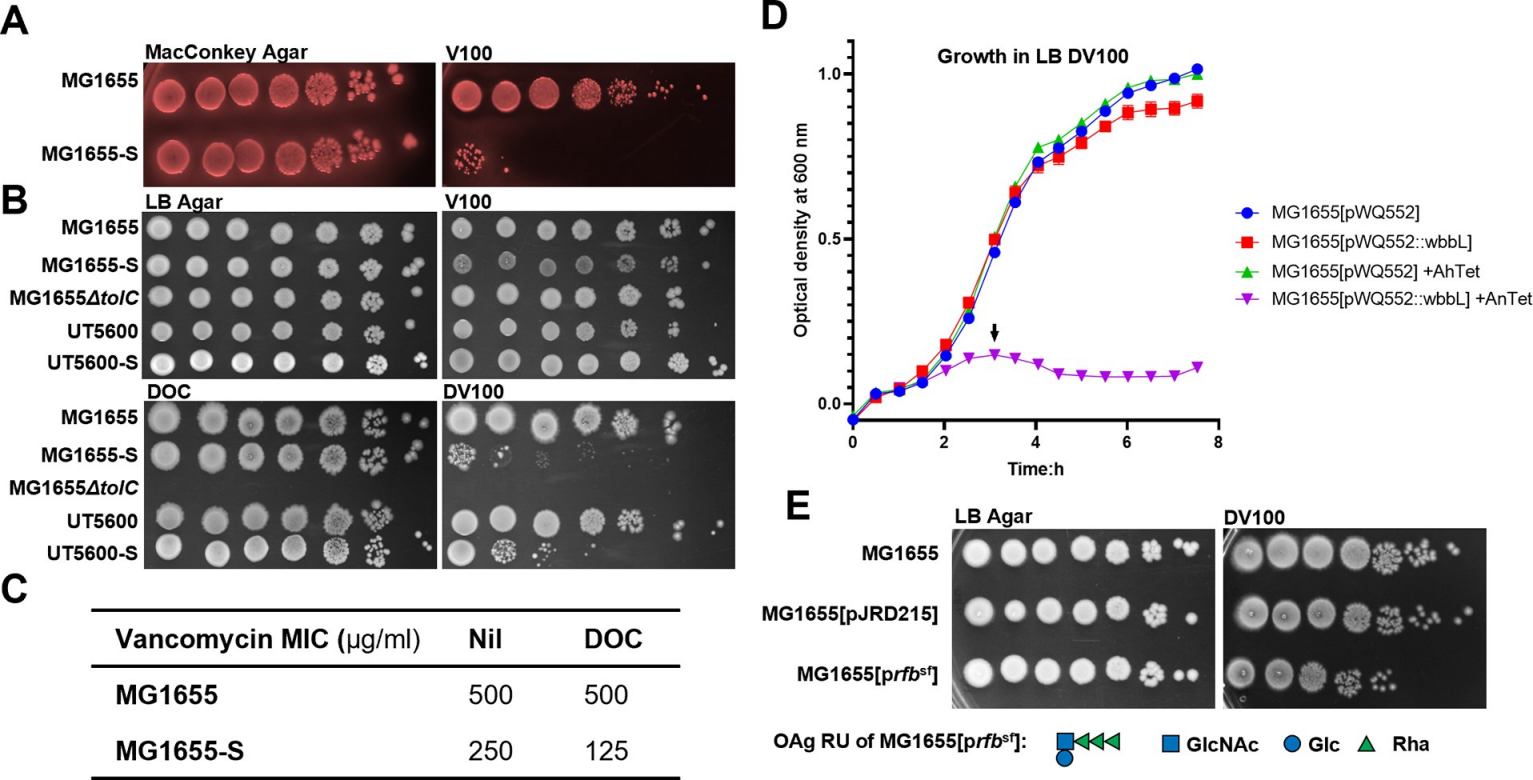
Restoration of O-antigen production sensitises *E*. *coli* K-12 to bile salts vancomycin. Bacterial cultures of indicated strains grown in LB media were adjusted to OD_600_ of 1 and spotted (4 μl) in 10-fold serial dilutions (10^0^ to 10^−6^) onto MacConkey agar (A) or LB agar (B & E) supplemented without or with 100 μg/ml vancomycin (V100), 0.1% (w/v) sodium deoxycholate (DOC), or both (DV100). p*rfb*^sf^, pJRD215 carrying *rfb* region from *Shigella flexneri*. The OAg RU structure produced in MG1655[p*rfb*^*sf*^] is shown. (C) Vancomycin minimum inhibitory concentration (MIC, μg/ml) for indicated *E*. *coli* strains in the absence (Nil) or presence of DOC. (D) Growth curves of indicated *E*. *coli* K-12 strains harbouring plasmids without or with *wbbL* cultured in LB media supplemented with DV100 and with or without 1 ng/ml anhydrotetracycline (AnTet). Arrow indicates lysis.

To examine the potential envelope stress posed by the commonly used MacConkey selective media to both MG1655 and MG1655-S, we supplemented MacConkey Agar with the antibiotic vancomycin, a drug that inhibits peptidoglycan (PG) synthesis by binding to D-Ala-D-Ala of PG precursors and is relatively impermeable for the intact *E*. *coli* outer membrane [25]. While MG1655 exhibited vancomycin resistance on MacConkey agar as expected, MG1655-S showed a dramatic increase in vancomycin susceptibility under these conditions (Fig 2A). The selective growth inhibition of most Gram-positive bacteria by MacConkey media is attributed to the presence of crystal violet and BS. However, supplementation of LB agar with BS alone (as sodium deoxycholate, DOC) (Fig 2B), but not crystal violet (S2A Fig) in a concentration that showed growth inhibition of a *tolC* mutant of MG1655 (Figs 2B and S2A), was sufficient to increase the susceptibility of MG1655-S towards vancomycin. Strikingly, despite MG1655-S exhibiting increased susceptibility to vancomycin in the presence of DOC compared to MG1655 (Fig 2C), no differences were detected in BS resistance (S2B Fig) or outer membrane leakage caused by DOC (S2C Fig) between the strains. Nevertheless, these data suggest that the production of OAg^O16^ in MG1655-S sensitised it to vancomycin in the presence of BS. Indeed, the expression of WbbL *in trans* in MG1655, which restores the production of OAg^O16^, in the presence of DOC and vancomycin resulted in bacterial lysis when cultured (Fig 2D). In addition, bacterial sensitivity to a combination of DOC and vancomycin (DV) in the presence of OAg^O16^ was not limited to strain MG1655, since OAg^O16^ restoration in K-12 strain UT5600 (S1F Fig) also sensitised bacteria towards DV (Fig 2B). Furthermore, production of *Shigella flexneri* 4a OAg type following introduction of the *S*. *flexneri rfb* region [26] into MG1655 also increased susceptibility to DV treatment (Fig 2E), suggesting that observed effects were not limited to the specific O16 structure and are likely due to OAg biogenesis.

### Disruption of OAg biogenesis in MG1655-S restores resistance to VBS

**Fig 3:**
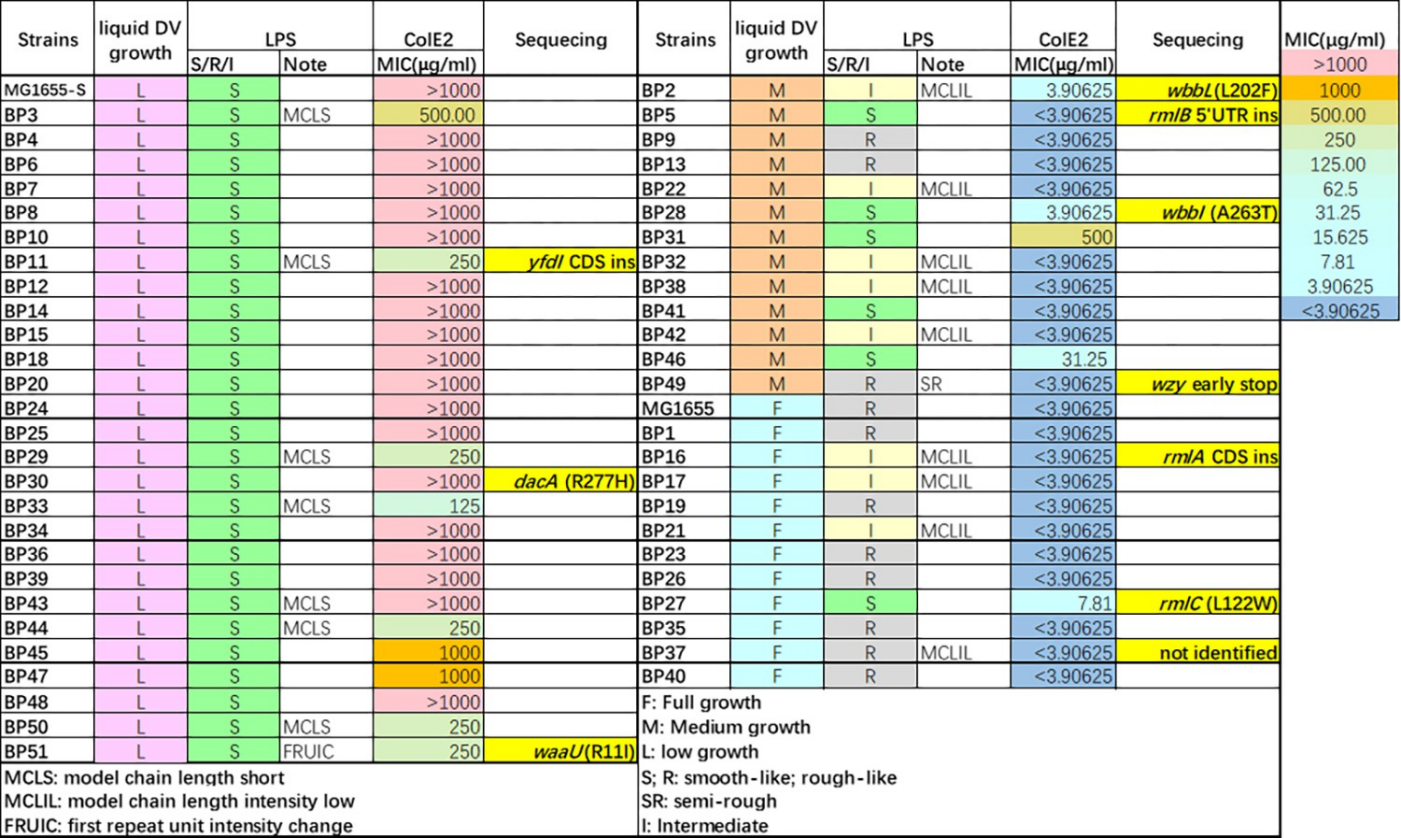
**Genotypic and phenotypic traits of isolated MG1655-S suppressor mutants.** Growth profiles of 51 MG1655-S suppressor mutants (BP1-51) in LB supplemented with 100 μg/ml vancomycin and 0.1% (w/v) sodium deoxycholate (DV) were classified as low (L) and similar to MG1655-S, medium (M) with growth recovery in between MG1655 and MG1655-S, or full (F) and similar to MG1655. The LPS silver staining profile of each mutant was compared to MG1655-S and MG1655 and classified as smooth-like LPS (S), rough-like LPS (R), or intermediately O antigen substituted LPS (I) with additional detailed alterations described in notes. The MIC values (μg/ml) of purified colicin E2 (ColE2) are recorded for each mutant. Suppressor mutants selected for whole genome sequencing are highlighted in yellow shading and their identified mutations are detailed by gene/coding sequence (CDS) name and non-synonymous substitution, early stop or insertion (ins). The mutation in BP49 was identified in *wzy* by targeted Sanger sequencing.

To investigate the mechanism of increased susceptibility to VBS in MG1655-S, we collected 51 independent suppressor mutants (BP1-51) recovered following MG1655-S growth on agar plates supplemented with DV. Culturing isolated suppressor mutants in plain LB medium exhibited no growth defects compared to MG1655-S or MG1655 (S3A Fig), yet mutant growth patterns in media supplemented with DV fell into three distinctive groups (Fig 3): i) early exponential growth followed by lysis similar to MG1655-S parent strain (low DV resistance growth group, termed **L**) (S3B Fig), ii) intermediate restoration of growth to mid-exponential phase and then stalled (medium DV resistance growth group, termed **M**) (S3C Fig), and iii) full growth similar to wild-type MG1655 (full DV resistance growth group, termed **F**) (S3D Fig). Strikingly, LPS profile analysis of suppressor mutants revealed a strong association between S-LPS production and susceptibility to DV (Figs 3 and S3E), whereby i) in the **L** group with the least increase in DV resistance, all (27/27) suppressor mutants maintained production of S-LPS, despite some observed alterations in either OAg modal length pattern or band intensity, ii) in the **M** group with intermediate increase in DV resistance, 8 out of 13 suppressor mutants had reduced S-LPS production, and iii) in the **F** group with fully restored resistance to DV, the majority (10/11) of suppressor mutants had undetectable or reduced S-LPS. We also used colicin E2 resistance to uncover any defects in O16-S-LPS that might be undetectable by silver staining. Strikingly, we found that over 60% (32/51) of all suppressor mutants had increased sensitivity to colicin E2 in comparison to the parent strain MG1655-S (Fig 3). Of those, the majority (24/32) showed medium (**M**) or full (**F**) growth restoration in media supplemented with DV. In contrast, most **L** group suppressors (18/27) exhibited the same colicin E2 resistance as the MG1655-S parent strain (Fig 3). Together, these data suggest that the production of O16-S-LPS reduces resistance to DV treatment, and that different mechanisms of suppressing VBS stress have been selected in the three groups of isolated MG1655-S suppressors.

To study the suppressor genotypes, we chose 3 representative mutants from each of the three DV growth recovery groups (**L**, **M**, and **F**) and analysed them by whole genome sequencing (Fig 3). We found that 7 of the 9 suppressor mutants had mutations in O16-S-LPS synthesis pathways. Specifically, mutations were identified in: (i) the glycosyltransferase *yfdI* gene responsible for glucose modification of OAg^O16^ in the periplasm [27] (BP11); (ii) in K-12 LPS outer core synthesis gene *waaU* (BP51); (iii) in genes required for the synthesis of OAg^O16^ in the cytoplasm (*wbbL* in BP2 and *wbbI* in BP28); and (iv) in genes responsible for dTDP-L-rhamnose synthesis (an OAg^O16^ sugar) (*rmlA* in BP16, *rmlB* in BP5 and *rmlC* in BP27). Suppressor mutant BP49, which showed an LPS profile with only one RU^O16^ substitution (S3E Fig), was confirmed to have a mutation in gene *wzy* encoding OAg polymerase by targeted sequencing of the *wzy* locus. In addition, in mutant BP30 we identified a mutation in *dacA*, that is a gene responsible for peptidoglycan carboxypeptidase, known as penicillin binding protein 5 (PBP5). Disruption of *dacA* was shown to have increased vancomycin resistance in MG1655 previously [28]. However, the isolated DacA^R227H^ mutation conferred the least DV resistance in BP30 (Fig 3), suggesting that the main stress posed by DV treatment on MG1655 is from the presence of DOC and is synergistically enhanced by vancomycin. Interestingly, we did not identify any genetic changes in the genome of BP37 by short read-based whole genome sequencing, which has limitations in detecting repeat expansions and large genomic rearrangement [29]. Nevertheless, BP37 showed full restoration of DV resistance and an altered LPS profile, suggesting that the alleviation of DV stress in this suppressor mutant is through the disruption of OAg biogenesis or assembly.

**Fig 4:**
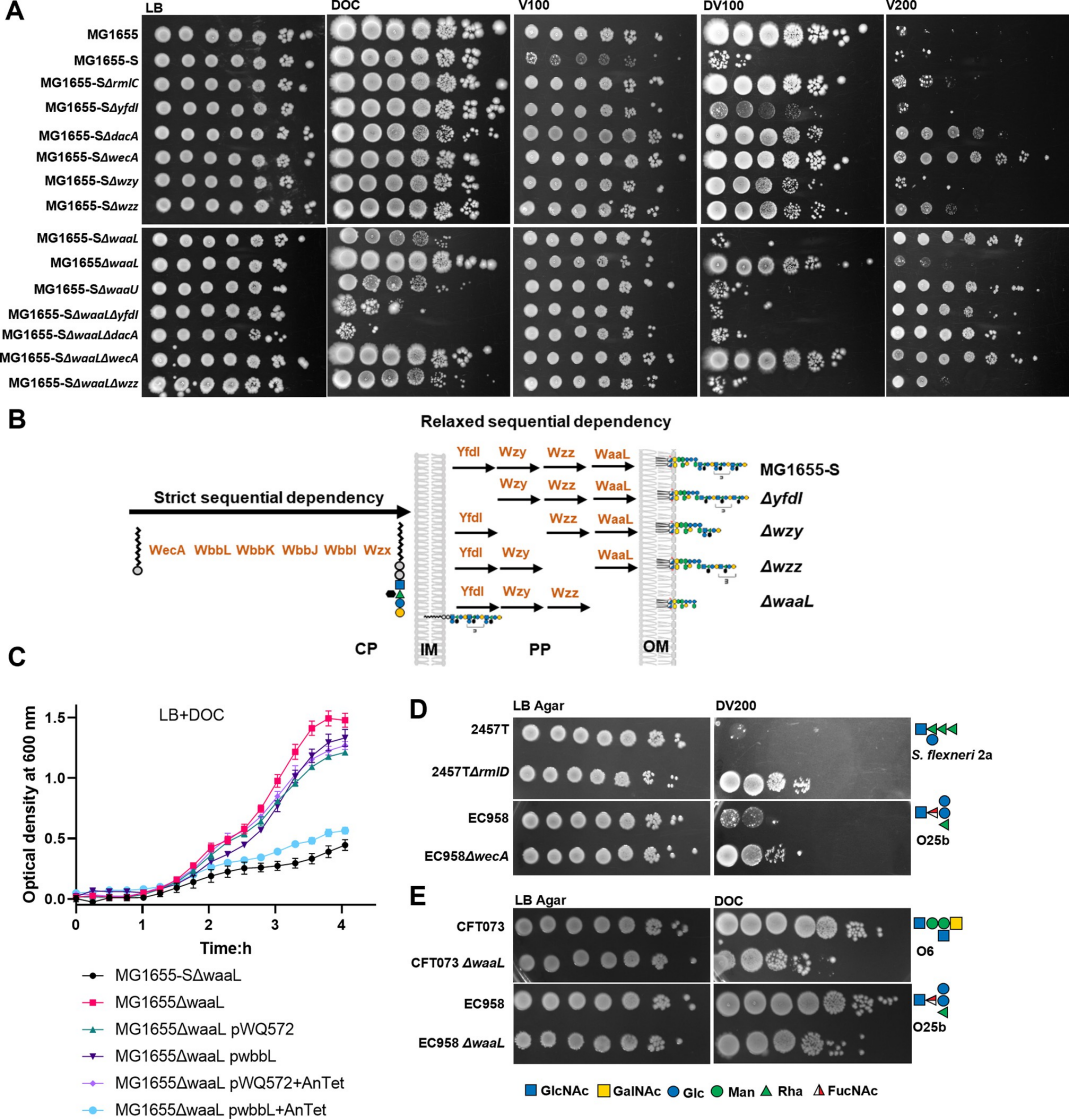
Accumulation of O antigen in the periplasm sensitises *E*. *coli* K-12 to bile salts. Bacterial cultures of indicated strains grown in LB media were adjusted to OD_600_ of 1 and spotted (4 μl) in 10-fold serial dilutions (10^0^ to 10^−6^) onto LB agar (A, D & E) without or supplemented with vancomycin at the concentrations of 100 μg/ml (V100) or 200 μg/ml (V200), 0.1% (v/w) sodium deoxycholate (DOC), a combination of V100 and DOC (DV100) or DOC and 200 μg/ml vancomycin (DV200). OAg RU structures of 2457T (*S*. *flexneri* 2a), EC958 (O25b), and CFT073 (O6) are shown. (B) Schematic representation of the synthesis and assembly process of O16 substituted LPS in different MG1655-S mutants. (C) Growth curves of indicated *E*. *coli* K-12 strains harbouring plasmids without or with *wbbL* cultured in LB media supplemented with 0.1%(w/v) DOC and without or with 1 ng/ml anhydrotetracycline (AnTet).

To validate suppressor mutant findings, we generated single gene deletion mutants of *rmlC*, *yfdI*, *dacA* and *wzy* in MG1655-S. The resulting strains showed restored resistance to DV treatment (Fig 4A), similar to isolated suppressor mutants. As expected, MG1655-S*ΔdacA* showed increased vancomycin resistance in comparison to MG1655-S (Fig 4A). Interestingly, MG1655-S*ΔrmlC* showed a slight increase in vancomycin resistance (Fig 4A). In addition, complementation by *rmlC*^WT^ but not *rmlC*^L122W^ (suppressor mutation in BP27) restored the production of S-LPS in MG1655-S*ΔrmlC* (S4A Fig) and re-sensitised it to DV (S4B Fig). Ectopic expression of RmlC^WT^ in BP27 also re-sensitised this suppressor mutant to DV (S4B Fig). These data confirmed that the suppressor mutations identified in genes involved in O16-S-LPS synthesis in MG1655-S are directly responsible for regaining of VBS resistance in these strains. Taken together, our genotypic analyses confirmed the phenotypic finding that biogenesis of O16-S-LPS in MG1655-S sensitised bacteria towards VBS treatment.

### Presence of polymerised C_55_-PP-OAg in the periplasm sensitises bacteria to bile salts

Disruption of OAg synthesis and/or assembly enhances production of enterobacterial common antigen (ECA) in *E*. *coli* due to the shared initial substrate C_55_-PP-GlcNAc that can be utilised both by the OAg^O16^ second glycosyltransferase WbbL and the ECA second glycosyltransferase WecG [30]. In addition, ECA has been reported previously to be important for DOC resistance [31]. However, ECA in MG1655-S mutants disrupted for OAg synthesis and/or assembly did not account for their increased resistance to DV since MG1655-S*ΔwecA* that has lost the production of both OAg and ECA (due to inability to synthesise the common precursor C_55_-PP-GlcNAc) (S4C Fig) also showed restored DV resistance (Fig 4A). Similar to MG1655*ΔwecA*, reported previously [32] to have increased resistance to vancomycin due to the loss of cyclic ECA, MG1655-S*ΔwecA* also exhibited elevated vancomycin resistance (Fig 4A). In addition, we also generated a single deletion of *wzz* mutant of MG1655-S (encodes the OAg co-polymerase responsible for modal chain length control) (S4C Fig). Interestingly, disruption of *wzz*, also alleviated DV susceptibility with a slight increase in vancomycin resistance (Fig 4A). Taken together, the inactivation of genes that catalyse processing steps of OAg^O16^ in the periplasm, such as *yfdI*, *wzy* and *wzz* (Fig 4A) restored resistance to DV, similar to disrupting genes responsible for OAg^O16^ synthesis in the cytosol (*rmlA*, *rmlB*, *rmlC*, *wbbL*, *wbbI*, *wecA*) (Figs 3 and 4A).

Synthesis of OAg^O16^ in the cytosol is strictly sequential (Fig 4B) as it depends on the distinct substrate specificity of each of the various glycosyltransferases. Synthesis of RU^O16^ will stall when either a glycosyltransferase or its sugar substrate is missing. On the contrary, the modification of OAg^O16^ (by YfdI), its polymerisation (by Wzy/Wzz) and ligation (by WaaL) in the periplasm have relaxed sequential dependency (Fig 4B) due to competition over universal substrates, where the absence of one does not impede the function of the rest. It is possible that disrupting the genes responsible for OAg cytosolic synthesis in MG1655-S completely abolished or delayed the accumulation of C_55_-PP-OAg^O16^ into the periplasm, while deletion of genes responsible for C_55_-PP-OAg^O16^ periplasmic processing accelerated its clearance in the periplasm (through translocation across the outer membrane by the Lpt apparatus [33]). To test this hypothesis, we generated the ligase mutant MG1655-S*ΔwaaL* (S4C Fig) that is unable to transfer OAg^O16^ from C_55_-PP onto the lipid A core oligosaccharide and would thus accumulate C_55_-PP-OAg^O16^ in the periplasm (S4D Fig) [34]. While disruption of *waaL* in MG1655 did not affect resistance to DV, in MG1655-S disruption of *waaL* failed to restore resistance to DV (Fig 4A) despite affecting LPS production (the mutant has a R-LPS profile, S4C Fig). Indeed, a lipid A core oligosaccharide mutant MG1655-S*ΔwaaU* that is unable to accept OAg^O16^ (S4C Fig) and would also accumulate C_55_-PP-OAg^O16^ in the periplasm (S4D Fig) also failed to restore resistance to DV (Fig 4A). These findings suggest that accumulation of C_55_-PP-OAg^O16^ in the periplasm sensitised the strain to DV. Intriguingly, susceptibility was due to increased sensitivity to DOC (Figs 4A and S4E) as both MG1655-S*ΔwaaL* and MG1655S*ΔwaaU* unexpectedly exhibited increased vancomycin resistance (Fig 4A). Similarly, restoration of OAg^O16^ production by WbbL expression in MG1655*ΔwaaL* slowed down its growth in the presence of DOC to the same level as MG1655-S*ΔwaaL* (Fig 4C), confirming that DOC exerts stress on the production of C_55_-PP-OAg^O16^ in the periplasm. To further explore this hypothesis, we generated double mutants in MG1655-S*ΔwaaL*. While deletion of *yfdI*, *dacA* and *wzz* rescues MG1655-S from DV stress, these gene deletions in MG1655-S*ΔwaaL* failed to restore DV resistance and bile salt resistance (Fig 4A). In contrast, deletion of *wecA* in MG1655-S*ΔwaaL*, which abolished the presence of C_55_-PP-OAg^O16^ (S4D Fig) in the periplasm, fully restored the strain’s DV resistance (Fig 4A). Interestingly, deletion of *wzz*, but not *yfdI*, *dacA* or *wecA*, abolished the elevated vancomycin resistance observed in MG1655-S*ΔwaaL* (Fig 4A), while deletions of *yfdI* and *dacA* in MG1655-S*ΔwaaL* further sensitised it to DOC (Fig 4A). We were unable to construct a MG1655-S*ΔwaaLΔwzy* mutant despite multiple attempts, presumably due to potential lethality caused by the accumulation of dead-end intermediates sequestering C_55_-P in the periplasm. However, deletion of *wzy* in a smooth strain would increase the intermediates pool of C_55_-PP-RU in the periplasm [34], yet MG1655-S*Δwzy* instead restored the VBS resistance (Fig 4A). This suggests that the chain length of OAg on the C_55_-PP-OAg in the periplasm plays a role in VBS sensitivity. Collectively, these data suggest a model where either accumulation or delayed clearance of polymerised C_55_-PP-OAg^O16^ in the periplasm sensitises MG1655-S towards BS.

### BS sensitisation to OAg biogenesis exists among other S-LPS-producing strains

Since *E*. *coli* K-12 has been heavily domesticated in the laboratory, we questioned whether sensitivity to DV can also be observed in other strains, including clinically relevant isolates. Surprisingly, we found that *S*. *flexneri* 2457T*ΔrmlD* with disrupted *S*. *flexneri* 2a-OAg rhamnose saccharide synthesis, and uropathogenic *E*. *coli* (UPEC) ST131 EC958*ΔwecA* with disrupted O25b-OAg and ECA synthesis all showed increased resistance to DV compared to the wild-type parent strains (Fig 4D). In addition, deletion of *waaL* in UPEC clinical isolates EC958 and CFT073 (O6-OAg) sensitised them to DOC (Fig 4E). These data support the effect observed in MG1655-S, where presence of C_55_-PP-OAg sensitises bacteria towards BS, is likely shared widely by other *E*. *coli* smooth strains and presumably can be extended to other S-LPS-producing Enterobacteriaceae.

### Prolonged exposure of MG1655-S to BS promotes loss of OAg

**Fig 5:**
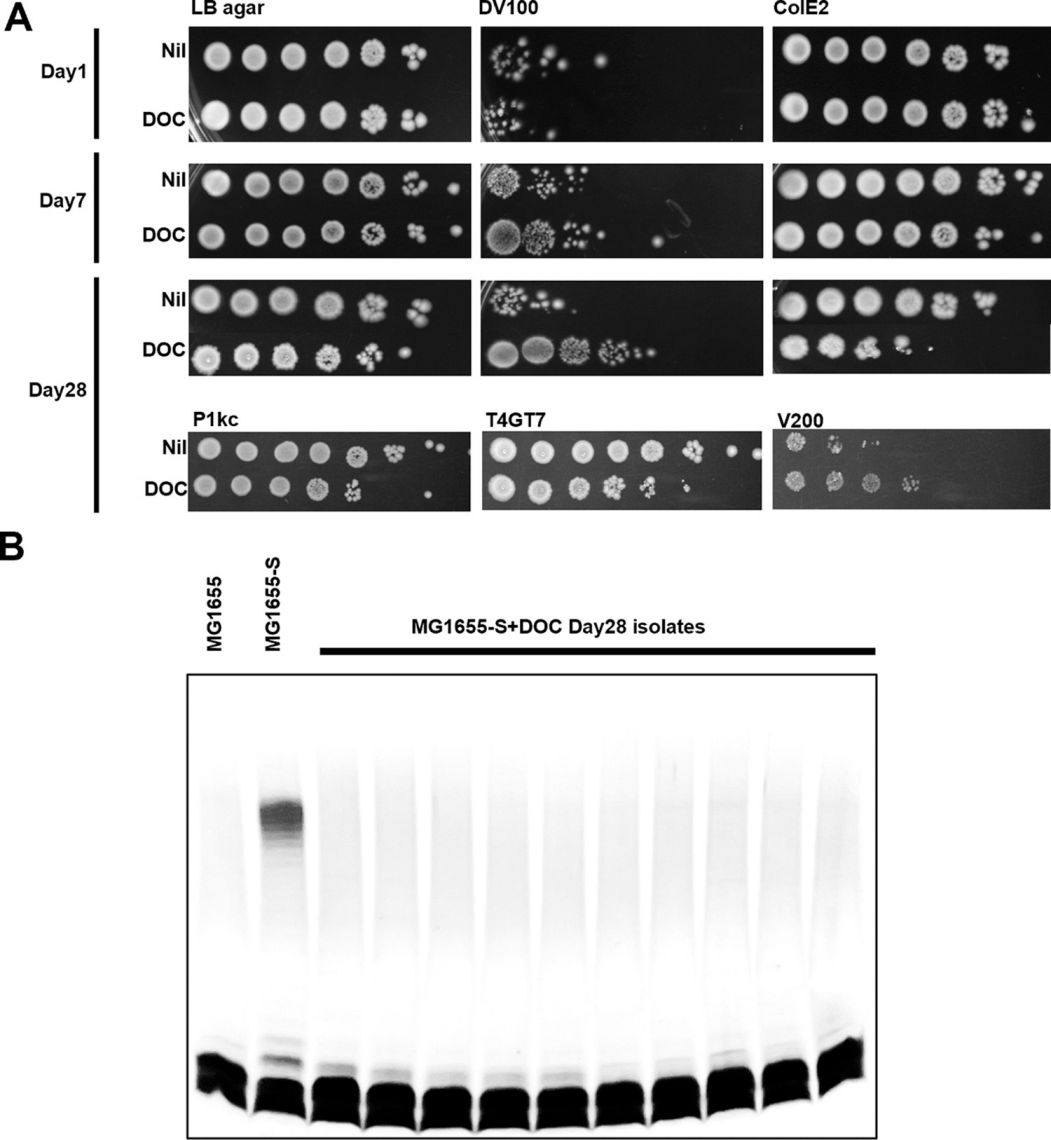
Continuous exposure of MG1655-S to bile salts promotes loss of O antigen. (A) Bacterial cultures of MG1655-S grown in LB media supplemented without (Nil) or with 0.1% (w/v) deoxycholate (DOC) for 1 day, 7 days or 28 days were adjusted to OD_600_ of 1, and spotted (4 μl) in 10-fold serial dilutions (10^0^ to 10^−6^) onto LB agar without or with 100 μg/ml vancomycin and 0.1% (w/v) sodium deoxycholate (DV100), 20 μg/ml crude colicin E2 preparation (ColE2), 200μg/ml vancomycin (V200), 200 μl of phage P1kc or T4GT7 lysate prepared from host MG1655 at the titre of above 10^10^ pfu. Data represent at least three independent repeats. (B) Silver staining of SDS-PAGE of LPS samples of MG1655, MG1655-S and its 28-day DOC-adapted isolates sensitive to colE2, P1kc and T4GT7.

Our results showed that disruption of OAg biogenesis in *E*. *coli* K-12 increased resistance to VBS, and that the stress is linked to the presence of polymerised C_55_-PP-OAg in the periplasm and BS. This suggests that loss of OAg in K-12 could be an adaptation to culture on BS-containing media. To test this tenet, we carried out an experimental evolution experiment where we continuously cultured MG1655-S in the presence or absence of DOC for up to 28 days and then challenged the cultures with both DV and colicin E2. At day 1 cultures showed no difference in DV resistance or colicin E2 susceptibility (Fig 5A). By day 7, DOC-treated MG1655-S showed a 10-fold increase in DV resistance and a 10-fold decrease in colicin E2 resistance compared to untreated MG1655-S (Fig 5A), strongly indicating that OAg biogenesis was disrupted in DOC-treated cells. Differences were further increased up to 1000-fold at day 28 (Fig 5A). Additionally, cultures of MG1655-S exposed to DOC for 28 days exhibited increased sensitivity to both phage P1kc and T4GT7, with both infecting K-12 isolates with R-LPS but not S-LPS [35]. Indeed, isolates that are sensitive to colicin E2, P1kc and T4GT7 all showed R-LPS phenotype by silver staining (Fig 5B). Intriguingly, the MG1655-S 28-day culture exposed to DOC also exhibited increased vancomycin resistance (Fig 5A), strongly suggesting an interference between OAg synthesis and cell wall synthesis upon bile salt exposure. Collectively, these data suggest that prolonged exposure of MG1655-S to BS can positively select for disruption of OAg synthesis.

## Discussion

Surface-exposed S-LPS is important for *E*. *coli* colonisation of host digestive tracts [8,36,37]. The OAg forms a protective surface layer around bacterial cells helping to withstand gut environmental challenge, including from host antimicrobial peptides (AMPs) [38] and gut-residing bacteriophages, such as the R-LPS-attacking coliphage T4 [39,40]. Originally isolated from human stool samples, *E*. *coli* K-12 strains however were found to be sensitive to coliphage T4 and unable to colonise the human gut [8]. This together with subsequent genetic evidence of independent inactivation of OAg^O16^ biogenesis in two *E*. *coli* K-12 strains originating from the same institute, strongly suggested that *E*. *coli* K-12 produced S-LPS upon isolation but was subjected to unknown selective pressure leading to early loss of OAg production. Here we investigated the potential reason for the loss of OAg among *E*. *coli* K-12 strains by focusing on its isolation and early maintenance in the 1920s. Bile salts, which are produced in the liver and released into the intestine, have been employed since the 1890s in bacterial culture as a key ingredient in selective media for the isolation and culturing of bacteria from human and animal faeces [20]. Indeed, *E*. *coli* can tolerate high concentrations of BS mainly due to the presence of an outer membrane that limits BS influx into cells, and by actively effluxing intracellular BS through the AcrAB-TolC pump [41]. Although BS are known to cause damage to membranes [42], DNA [43], and proteins [44], the exact mechanism of BS susceptibility in Gram-positive and Gram-negative bacteria with defective efflux pumps [45] still remains unclear.

Here we showed that while production of OAg in *E*. *coli* K-12 did not affect resistance to BS, it increased sensitivity to VBS. Since BS treatment indistinguishably caused outer membrane leakage in both MG1655 and MG1655-S, the increased sensitivity to vancomycin observed in MG1655-S is therefore likely not a direct result of outer membrane disruption. Indeed, in the absence of BS, MG1655-S exhibited a 2-fold increase in sensitivity to vancomycin in comparison to MG1655. Vancomycin effects are well studied in Gram-positive bacteria, where it’s been shown to bind to D-Ala-D-Ala at the terminus of pentapeptide linked peptidoglycan (PG) [46] and inhibit the transpeptidation of PG catalysed by penicillin binding proteins (PBPs). Our results in *E*. *coli* K-12 hence suggest two possible causes: i) a complex interplay may exist between biogenesis of OAg and PG in Gram-negative bacteria, where the production of OAg potentially interferes with the biosynthesis and/or maintenance of PG, which may lead to altered accessibility or availability of PG precursors to the limited amount of vancomycin that penetrates across the outer membrane, and ii) the biogenesis of OAg may compromise the bacterial cell envelope barrier which allows more entrance of vancomycin. The former possibility is supported by the evidence where the restored production of OAg in *E*. *coli* K-12 strains restored the morphological abnormalities caused by deletion mutations in PBPs [47].

We also observed a synergistic effect between BS and vancomycin in inhibiting the growth of MG1655-S but not MG1655. Synergistic effects were also seen in UPEC and *S*. *flexneri* wild-type strains (naturally producing S-LPS) but not in isogenic mutants with disrupted OAg synthesis, suggesting that our observations in *E*. *coli* K-12 are true more widely among the Enterobacteriaceae. Synergy of BS and vancomycin was also previously reported in other *E*. *coli* strains and an *Acinetobacter lwoffi* strain [48]. The synergy mechanism between BS and vancomycin against strains producing OAg remains unclear, as the target(s) of BS in *E*. *coli* remains unknown. Through our MG1655-S suppressor mutant studies, we found that most suppressors had defects in OAg synthesis pathways. Disruption of early OAg synthesis steps in the cytosol (*rmlABCD* and *wbbL*) can be expected to confer resistance to VBS, as it completely abolishes the use of C_55_-PP-GlcNAc in OAg synthesis and eliminates any potential interference with PG synthesis. This is exemplified by the *rfb-50* and *rfb-51* mutations in EMG2 and WG1, respectively. The synthesis of both PG and OAg requires the universal lipid carrier C_55_-P, thus an interference likely exists between the two pathways through competition for this common lipid carrier. However, this seems not to be the primary cause for the increased sensitivity of MG1655-S to VBS. This is because deletion of *wzy* in smooth strains has been shown to accumulate C_55_-PP-RU in the periplasm and sequestered a significant amount of C_55_-P in comparison to WT [34], with an impact on PG synthesis illustrated by changes in cell sizes [49]. However, the deletion of *wzy* in MG1655-S instead restored the resistance to VBS. Nevertheless, in addition to the *wzy* deletion mutant, we also observed increased resistance to VBS among mutants with disrupted YfdI, Wzz, which are IM-bound periplasmic proteins responsible for later processing of OAg in the periplasm, and which compete for C_55_-P-OAg^O16^ with the WaaL ligase. It is possible that competition between these proteins over C_55_-PP-OAg^O16^ limited the rate of polymerised O16 release from C_55_-PP by WaaL in the periplasm, resulting in increased bacterial sensitivity to VBS. Indeed, while single deletion of *yfdI*, *dacA* and *wzz* restores resistance to VBS in the MG1655-S background, it fails to restore it in MG1655-S*ΔwaaL*. This strongly supports our model that the accumulation of polymerised C_55_-PP-OAg in the periplasm renders the strain sensitive to VBS, primarily due to the sensitivity to BS and regardless of loss of RU side chain glucosyl modification by deleting *yfdI*, loss of modal chain length by deleting *wzz*, or increased vancomycin resistance by deleting dacA. The MG1655-S*ΔwaaLΔwecA* on the contrary, restored the resistance to VBS and BS, in agreement with results shown by MG1655-S*ΔwaaL*, CFT073*ΔwaaL* and EC958*ΔwaaL*, and MG1655*ΔwaaL*[pwbbL] that the production of C_55_-PP-OAg is the primary cause of increased sensitivity to both VBS and BS. In contrast, MG1655-S*Δwzy* which accumulates C_55_-PP-RU in the periplasm was not sensitive to BS, suggesting that the periplasmic polymerised RUs, but not single RU sensitises MG1655-S towards BS.

We have also observed increased vancomycin resistance upon disruption of *rmlC*, *dacA*, *wecA*, *wzz* and *waaL*, but not *yfdI* and *wzy* in MG1655-S. Disruption of the D,D-carboxypeptidase DacA (PBP5) in MG1655 was previously reported to increase vancomycin resistance [28] and here in MG1655-S in the presence of BS, and was proposed to be through producing increased decoy binding targets to vancomycin. The disruption of *wecA* would abolish the synthesis of cyclic ECA which has been demonstrated to have increased vancomycin resistance [32]. This could in part explain the slightly elevated vancomycin resistance in MG1655-S*ΔrmlC*, since the disruption of *rmlC* would accumulate increased level of dTDP-4-keto-6-deoxy-D-Glc, which may affect homeostasis of the saccharide substrate pool for making ECA. Disruption of *wzz* has been shown to have effects in ECA modality through potential cross-interaction between WzzE by Wzy [50] when missing the native co-polymerase partner Wzz, which could in turn alter vancomycin resistance through affecting ECA biogenesis. The elevated vancomycin resistance in MG1655-S*ΔwaaL* and MG1655-S*ΔwaaU* is however completely unexpected. Interestingly, although deletion of *wzz* slightly increased vancomycin resistance in MG1655-S, it drastically reduced vancomycin resistance in MG1655-S*ΔwaaL*, suggesting that the accumulated periplasmic OAg intermediate at the modal chain length is primarily responsible for the elevated vancomycin resistance in MG1655-S*ΔwaaL*. Strikingly, in the absence of vancomycin, exposure to BS alone selected for mutants with increased vancomycin resistance while encouraging the inactivation of OAg production. This again suggests an intricate interplay between OAg and PG synthesis pathways or cell envelope integrity as discussed earlier. However the exact nature of this interplay remains to be determined in future work currently underway. Based on our findings, we propose a model for explaining how *E*. *coli* K-12 lost its OAg, whereby the production of OAg generates transient polymerised C_55_-PP-OAg intermediate in the periplasm, and upon prolonged exposure to BS this enhances interference with cell wall synthesis pathways and growth inhibition which can be alleviated by disruption of OAg synthesis.

Regardless of the complex action of VBS in selecting MG1655-S mutants with disrupted OAg biogenesis, we were able to show that the stress posed by BS on MG1655-S was aided by vancomycin killing. A common way of preserving bacterial strains in the past was through stab agar culture (master culture) maintained at room temperature [4]. This practice potentially accelerated strain in-lab adaptation due to starvation in these long-term cultures [51]. However, here we showed that static culture of a genetically restored OAg^O16^ producing *E*. *coli* K-12 (MG1655-S) at room temperature for as long as 28 days did not select for differences in sensitivity to VBS, colicin E2, bacteriophage P1kc and T4GT7, suggesting that in-lab starvation alone could not pose adequate selection pressure for MG1655-S to lose its OAg production. Only when we cultured MG1655-S in commonly used media containing BS, could we select for characteristics of OAg biosynthesis disruption in over 90% of the culture population only within 1 week. It is likely that the selection pressure posed on MG1655-S in our in-lab adaptation experiments was a combination of the effects posed by BS on the transient presence of C_55_-PP-OAg in the periplasm discussed above, and the oxidative stress of exposure to long-term rich media [52].

Another widely used laboratory strain named *E*. *coli* B by Delbrück and Luria used in their early work studying interactions between bacteria and T even phage [53] was also devoid of OAg since the production of OAg was shown to abolish T even phage infections [35]. The history of B strain can be traced back to 1917 [54] where it was also used for reporting bacteriophage work [55]. Two famous B strain derivatives REL606 and BL21(DE3), which are still widely used for long-term evolution experiments and expression of recombinant proteins, respectively, were both sequencing confirmed [56] to have disruptions in both their OAg^O7^ biogenesis due to the inactivation of *wbbD* encoding the second glycosyltransferase for synthesising OAg^O7^, and LPS R1 core synthesis due to the inactivation of *waaT*, resulted in a truncated LPS outer core. Although it is unknown how the *E*. *coli* B strain lost its O antigen production, the inactivation of *waaT* may not be a direct result of BS adaptation, since LPS core truncation in both a rough background (as shown previously [57]) and in a smooth background with OAg intermediate accumulation in the periplasm (shown here) are sensitive to BS. It is possible that different mutational events are responsible for these two genotypes. Our findings clearly demonstrate a role for BS in disrupting OAg production in *E*. *coli* K-12 and other Enterobacteriaceae and would argue against routine use of MacConkey and other BS-containing media for prolonged cultures in bacteriology. Their value lies as secondary indicative media in aiding the identification of Enterobacteriaceae from faecal samples. While we are not in a position to trace the exact history of isolation, maintenance and manipulation of the original *E*. *coli* K-12 strain, or whether BS was definitively used during the passages of *E*. *coli* K-12 cultures for strain purification, our study provides for the first time the most likely explanation of how the original *E*. *coli* K-12 lost its OAg.

## Materials and methods

### Bacterial strains, plasmids and growth media

The bacterial strains, plasmids used in this work are listed in S1 Table. Single colonies of *E*. *coli* strains grown overnight on lysogeny broth (LB) (Bacto Tryptone (#211699, Gibco) 10g/L, Bacto Yeast Extract (#212750, Gibco) 5g/L, NaCl 5 g/L) [58] agar (1.5% w/v) plates were picked and grown overnight in LB at 37°C for all experiments. Other bacterial growth media used in this study were Mueller Hinton (#275730, BD), MacConkey Agar (#CM0115, Oxiod). Where appropriate, media were supplemented with ampicillin (Amp, 100 μg/ml), kanamycin (Kan, 50 μg/ml), chloramphenicol (Chl, 25 μg/ml), bile salts sodium deoxycholate (DOC, 0.1% w/v, #D6750, Sigma), vancomycin (Van, 100 μg/ml, #SBR00001, Sigma), L-arabinose (0.2% w/v), D-glucose (0.2% w/v), crystal violet (0.0005% w/v), and colicin E2 (20 μg/ml).

### Bacterial phage and phage lysate preparation

Bacteriophage P1kc (ATCC 11303-B23) and T4GT7 (kindly gifted by A/Prof. Keith Shearwin, The University of Adelaide) lysates were prepared by adding 100 μl of mid-exponential culture of MG1655 and phage stocks at a multiplicity of infection (MOI) of ~0.5 to 3ml of LB soft agar (0.75% w/v agar) supplemented with MC salts (100 mM MgSO_4_ and 5 mM CaCl_2_) and poured onto LB agar plate followed by incubation at 37°C for 18h. The clear top soft agar layer containing the phage lysate was carefully scrapped and mixed with 2 ml of LB-Miller media (LB supplemented with MC salts), vortexed and centrifuged. The clear media containing bacteriophage were collected and sterilised by 10 μl of chloroform. Phage titres were determined by spotting 5 μl of 10-fold serially diluted phage stock onto top LB soft agar plates containing 100 μl of MG1655 mid-exponential culture. Plaque forming units (pfu) were calculated after 18 h incubation of phage infected plates at 37°C.

### Site-directed mutagenesis via allelic exchange

*E*. *coli* gene inactivation mutants were generated by Lambda Red mutagenesis as described previously [59] with the oligos listed in S1 Table. For restoration of *wbbL* in K-12 strains MG1655 and UT5600, an amplicon containing the undisrupted WbbL coding sequence from pPR2191 [60] was generated by PCR with the oligos listed in S1 Table, and was then used for allelic exchange gene replacement as above. Successful revertants were selected on LB agar supplemented with 20 μg/ml purified colicin E2 protein. Removal of the IS5I elements in the original *wbbL* gene was confirmed via PCR and phenotypic restoration of O16 substituted LPS was confirmed with SDS-PAGE LPS silver staining. Whole genome sequencing of the resulting MG1655-S strain by DNBSEQ confirmed the successful removal of IS5I in *wbbL* without the introduction of any other genetic modifications.

### Plasmid construction

The RmlC and WaaL expression constructs used in this study were constructed by PCR amplification of the *waaL* and *rmlC* alleles from strains MG1655 (*rmlC*^WT^) and BP27 (*rmlC*^L122W^) using the Q5 high-fidelity polymerase (NEB) and oligos listed in S1 Table and subsequent insertion into pSU2718 and pBAD18-Chl, respectively, via restriction digestion cloning.

### RNase I leakage assay

RNase I leakage assay was done exactly as described previously [61]. Briefly, bacterial strains were streaked onto LB agar plates and grew at 37°C overnight. The plates were overlaid with 3 ml of LB soft agar (0.75% (w/v) agar) containing 1% (w/v) yeast RNA. After incubation at 42°C for 2 hr to permit digestion, plates were flooded with 0.1 M HCl to precipitate undigested RNA.

### Suppressor mutant selection

Suppressor mutant selection of MG1655-S to vancomycin and deoxycholate combination treatment was done by spreading an overnight MG1655-S culture grown in LB (1 in 2000 dilution in fresh LB media) onto LB agar supplemented with 100 μg/ml vancomycin and 0.1% (w/v) sodium deoxycholate, and subsequent incubation at 37°C overnight. Colonies were carefully streaked out onto non-selective LB plates and stored at -80°C for downstream analyses.

### Whole genome sequencing

For bacterial whole genome sequencing, genomic DNA of MG1655, MG1655-S and suppressor mutants was prepared using a Qiagen QIAamp DNA blood mini kit according to the manufacturer’s protocol. Samples were prepared for DNBseq DNA library construction (BGI) followed by DNBSEQ PE150 sequencing (BGI). The processed reads for each strain were then mapped onto the NCBI MG1655 reference genome (Accession number U00096) using Geneious 8.0. Sequence changes between our laboratory MG1655 and the online reference genome, as well as within-population variations were determined using the Geneious built-in function and are excluded from the analysis in MG1655-S and its suppressor mutants (S2 Table).

### Vancomycin and bile salts susceptibility assays

For vancomycin susceptibility testing, overnight cultures of bacterial strains grown in LB were adjusted to OD_600_ of 1 and spotted (4 μl) in 10-fold serial dilutions onto MacConkey agar supplemented with or without vancomycin at different concentrations. Culture dilutions were also spotted onto LB agar supplemented without or with vancomycin, deoxycholate, and crystal violet at different concentrations, as indicated in figure legends. Plates were incubated overnight at 37°C and then imaged.

### Colicin E2 purification and susceptibility assay

Colicin E2 protein was purified and used to determine the susceptibility of bacterial strains as described previously [24]. Briefly, C-terminal his-tagged colicin E2 protein with its immunity protein were expressed from BL21(DE3) carrying pET41b-ColE2 [62] and purified by immobilised metal affinity chromatography (IMAC) using Profinity Ni-charged resin (Bio-Rad) according to manufacturer’s protocol. For colicin E2 susceptibility assays, strains growing to mid-exponential phase (OD~0.5) were spread onto LB agar, and a 5 μl of purified colicin E2 diluted in 2-fold series in PBS was spotted onto the plate. Plates were incubated overnight at 37°C for 18 h and the sensitivity level was determined by the minimum colicin E2 concentration that showed clear bacterial growth inhibition.

### Bacterial growth assays

Growth curves for bacterial strains were generated as described previously [63]. Briefly, bacterial strains grown overnight in LB were diluted 1 in 1000 in 200 μl of LB in flat-bottom 96 well plates supplemented without or with 0.1% (w/v) sodium deoxycholate and/or 100 μg/ml vancomycin. For expression of plasmid-borne *wbbL* under *tet* promoter control, 1 ng/ml anhydrotetracycline was also supplemented in media. Microtiter plates were incubated at 37°C with aeration in a CLARIOstar plate reader (BMG, Australia) programmed to measure the optical density (O.D. 600 nm) every 6 minutes over 18 h.

### Antibiotic susceptibility testing

Vancomycin minimum inhibitory concentrations (MIC) were determined for bacterial strains according to the Clinical and Laboratory Standards Institute guideline [64]. Briefly, overnight bacterial cultures in LB were sub-cultured in MH broth to a McFarland turbidity standard of 0.5. Cultures were then adjusted to 10^7^ cells/ml and 10 μl samples were spotted onto MH agar plates supplemented with vancomycin at concentrations from 500 μg/ml to 7.8 μg/ml in 2-fold serial dilutions in the presence or absence of 0.1% (w/v) sodium deoxycholate. Plates were incubated overnight at 37°C. The MIC value was determined as the lowest concentration of vancomycin at which bacterial lawn growth was inhibited.

### *In vitro* adaptation to bile salts

MG1655-S grown overnight at 37°C in LB broth was sub-cultured 1:1000 in 3 ml LB broth in a 15 ml tube supplemented with or without 0.1% (w/v) sodium deoxycholate in triplicate. Tube lids were loosely attached to allow aeration and tubes were incubated at 21°C statically for 1 day, 7 days and 28 days. Cultures from different time-points were 10-fold serially diluted and spotted onto LB agar plates supplemented with or without 100 μg/ml vancomycin and 0.1% (w/v) sodium deoxycholate, 20 μg/ml colicin E2, or 200 μg/ml vancomycin, or plates pre-spread with 200 μl of either phage P1kc or T4GT7 at the titre above 10^10^ pfu. Plates were incubated overnight at 37°C and imaged.

### LPS silver staining

Bacteria cells (10^9^ cells) grown at mid-exponential phase were harvested via centrifugation (20,000 g, 1 min) and lysed in 50 μl of SDS sample buffer and heated at 100°C for 10 min. Lysates were then cooled to room temperature and treated with 50 μg/ml proteinase K (PK, NEB) for 18 h at 60°C. PK treated samples were then heated again at 100°C for 10 min, and 2–5 μl of each sample was loaded onto 10–20% SDS-tricine gels (Invitrogen, #EC66252BOX) and LPS was silver stained exactly as described previously [65]. Wherever needed, polysaccharide samples separated by SDS-tricine gel electrophoresis were then transferred onto nitrocellulose membrane and detected with rabbit polyclonal anti-O16 antibodies (SSI Diagnostica, #SSI85012).

## Supporting information

S1 Table Strains, plasmids, and oligonucleotides. (PDF)

S2 Table Differences of MG1655 used in this study and reference MG1655 (U00096). (PDF)

S1 Fig Restoration of O16 production in *E*. *coli* K-12 strains. (A) Schematic representation of the strategy employed for *wbbL* allelic replacement in the *rfb* gene cluster in MG1655 to construct strain MG1655-S. The respective O16 repeating units that would be produced in the cytosol denote the expected LPS products by each strain. Primers P1 and P2 used to generate MG1655-S are mapped onto *rfb* regions by black arrows. (B) PCR amplicons of *wbbL* region from MG1655 and MG1655-S (using primers P3 and P4) confirming the replacement of an intact *wbbL* CDS in MG1655-S. (C) Colicin sensitivity assay confirming increased resistance of MG1655-S due to regained O antigen substituted LPS production. Colicin E2 was used at 1 mg/ml and in subsequent 2-fold dilutions (5 μl spots). (D&F) Silver stained SDS-PAGE of LPS samples from two sets of *E*. *coli* K-12 strains (wild-type MG1655 and UT5600 carry the IS51 element in *wbbL* and MG1655-S/UT5600-S are isogenic *wbbL* intact strains, respectively). LPS patterns confirm restored production of O antigen substituted LPS in the engineered MG1655-S and UT5600-S strains. (E) Western immunoblotting of samples as in (D) with anti-O16 antibodies showing the restoration of O16 O antigen production in MG1655-S.
(TIF)

S2 Fig The effect of crystal violet and DOC on *E*. *coli* K-12 MG1655 and MG1655-S. (A&B) Bacterial cultures of indicated strains grown in LB media were adjusted to OD_600_ of 1 and spotted (4 μl) in 10-fold serial dilutions (10^0^ to 10^−6^) onto LB agar supplemented with 0.0001% (w/v) crystal violet (CV) or 100 μg/ml vancomycin and CV (CVV100), or 5% and 2.5% (w/v) sodium deoxycholate (DOC). (C) RNase I leakage assay of MG1655 and MG1655-S grown on LB agar supplemented without or with 0.1% (w/v) DOC.
(TIF)

S3 Fig Phenotypic analyses of 51 MG1655-S suppressor mutants. Growth curve of MG1655, MG1655-S and BP1-BP51 suppressor mutants in LB media (A) or in LB supplemented with (B-D) 100 μg/ml vancomycin and 0.1% (w/v) DOC (DV100). E) Silver staining of SDS-PAGE of LPS samples prepared from MG1655, MG1655-S and BP1-BP51 suppressor mutants.
(TIF)

S4 Fig Effect of DOC and vancomycin on MG1655-S mutants. (A & C) Silver staining of SDS-PAGE of LPS samples of MG1655, MG1655-S and their mutational derivatives. Supplementation of media with 0.2% (w/v) arabinose was used in samples as indicated (+ara). Bacterial cultures of indicated strains (B & E) grown in LB media were adjusted to OD_600_ of 1 and spotted (4 μl) in 10-fold serial dilutions (10^0^ to 10^−6^) onto LB agar supplemented without or with 0.1% (w/v) DOC and 100 μg/ml vancomycin (DV100) in the absence or presence of 0.2% (w/v) L-arabinose (DV100ara). (D) Western immunoblotting of proteinase K-treated LPS samples with anti-O16 antibodies showing OAg^O16^-capped LPS in MG1655-S (red dashed box) and the accumulated C55-PP-OAg^O16^ intermediates (green dashed box) in MG1655-S *ΔwaaL* and *ΔwaaU* mutants.
(TIF)
