# Supplementary material for: O antigen biogenesis sensitises Escherichia coli K-12 to bile salts, providing a plausible explanation for its evolutionary loss
Source: PLoS Genet. 2023 Oct 4;19(10):e1010996. doi: 10.1371/journal.pgen.1010996 (PMC10578602; doi:10.1371/journal.pgen.1010996)
Supplement: S1 Table — (PDF) [file pgen.1010996.s001.pdf]

S1 Table. Strains, plasmids, and oligonucleotides

Bacterial strains

**Strains**	**Description**	**Source**
MG1655	Wild-type *E. coli* K-12 MG1655	Lab stock
MG1655*ΔwaaL*	MG1655*ΔwaaL::kan*	This work
MG1655*ΔtolC*	MG1655*ΔtolC::chl*	This work
MG1655-S	MG1655 with IS5I removed in *wbbL*	This work
MG1655-S*ΔrmlC*	MG1655-S*ΔrmlC::kan*	This work
MG1655-S*ΔwecA*	MG1655-S*ΔwecA::kan*	This work
MG1655-S*Δwzy*	MG1655-S*Δwzy::chl*	This work
MG1655-S*Δwzz*	MG1655-S*Δwzz::chl*	This work
MG1655-S*ΔyfdI*	MG1655-S*ΔyfdI::kan*	This work
MG1655-S*ΔdacA*	MG1655-S*ΔdacA::kan*	This work
MG1655-S*ΔwaaL*	MG1655-S*ΔwaaL::kan*	This work
MG1655-S*ΔwaaLΔyfdI*	MG1655-S*ΔwaaL::frt ΔyfdI::kan*	This work
MG1655-S*ΔwaaLΔwzz*	MG1655-S*ΔwaaL::frt Δwzz::kan*	This work
MG1655-S*ΔwaaLΔdacA*	MG1655-S*ΔwaaL::frt ΔdacA::kan*	This work
MG1655-S*ΔwaaLΔwecA*	MG1655-S*ΔwaaL::frt ΔwecA::kan*	This work
MG1655-S*ΔwaaU*	MG1655-S*ΔwaaU::kan*	This work
BP1-BP51	Library of 51 independent suppressor mutants of MG1655-S	This work
UT5600	*E. coli* K-12, *ompT*	[1]
UT5600-S	UT5600 with IS5I removed in *wbbL*	This work
2457T	*Shigella flexneri* 2a, 2457T	[2]
2457T*ΔrmlD*	2457T *ΔrmlD::kan*	[3]
EC958	Uropathogenic *E. coli* cystitis isolate	[4]
EC958*ΔwaaL*	EC958*ΔwaaL::chl*	This work
EC958*ΔwecA*	EC958*ΔwecA::chl*	This work
CFT073	Uropathogenic *E. coli* pyelonephritis isolate	[5]
CFT073*ΔwaaL*	CFT073*ΔwaaL::chl*	This work
TOP10	F–mcrA Δ(mrr-hsdRMS-mcrBC) φ80lacZΔM15 ΔlacX74 recA1 araD139 Δ(ara-leu)7697 galU galK λ– rpsL(StrR) endA1 nupG	Invitrogen

Plasmids

**Plasmids**	**Description**	**Source**
pSU2718	Cloning plasmid, lac promoter, Chl^R	[6]
pBAD18cm	Cloning plasmid, arabinose promoter, Chl^R	[7]
pKD46	Temperature sensitive plasmid expressing Red proteins, Amp^R	[8]
pKD4	Plasmid carrying FRT flanked kanamycin resistant cassette, Amp^R, Kan^R	[8]
pKD3	Plasmid carrying FRT flanked kanamycin resistant cassette, Amp^R, Chl^R	[8]

pCP20	Temperature sensitive plasmid carrying FLP flippase, Amp^R	[8]
pWQ572	Tetracycline inducible promoter, Chl^R	[9]
pWbbL	*wbbL* CDS cloned from WG1 into pWQ572	[10]
$pRmlC^{WT}$	*rmlC* cloned from MG1655 into pBAD18cm	This work
$pRmlC^{L122W}$	$rmlC^{L122W}$ cloned from BP27 into pBAD18cm	This work
pWaaL	*waaL* cloned from MG1655 into pSU2718	This work
pJRD215	Cosmid, Kan^R	[11]
$prfb^{sf}$	pJRD215 carrying *rfb* region from *S. flexneri*, pRMA154	[12]

Oligos

**Description**	**Sequence**
wbbL CDS F/P1	ATGGTATATATAATAATC
wbbL CDS R/P2	TTATATTACGGGTGAAAA
wbbL screening F/P3	ATTGACTTCAAAAAAGGTAACCTC
wbbL screening R/P4	GAATGTTCGCAAATAAGTATACAAAG
tolC KO F	CCTTTTGCGGTAGCGGCTTCTGCTAGAATCCGC AATAATTTTACAGTGTAGGCTGGAGCTGCTTC
tolC KO R	CTCGCTGGCACCAACAAAGTTGTACTGGGCCTG TTTCACCATGGGAATTAGCCATGGTCC
wzy KO F	CGCTCTTTATCAAGTGAAAAATATAATGAGTAC GGATTAAGTGTAGGCTGGAGCTGCTTC
wzy KO R	CGCGTCTAGAGAAATTTAAATCATTCAAAAAAT ACATTTTATGGGAATTAGCCATGGTCC
wzz KO F	AACTATGCGGACTTGGAAATTTCCGTCAGTTAG GGTAATGGTGTAGGCTGGAGCTGCTTC
wzz KO R	GGTGTCACCACCCTGCCCTTTTTCTTAAAACCG AAAAGAATGGGAATTAGCCATGGTCC
yfdI KO F	CAATAAAAGTATCATTGTATATATCTTTTGTTTT GATTATTTGCGCCTTAGTGTAGGCTGGAGCTGC TTC
yfdI KO R	ACATCGCTATTGTCTTGATTTCTTTGAATTATTT TATAAATTAAAAAAACATGGGAATTAGCCATGG TCC
dacA KO F	TTTTTTCCGCTCGTATCATGAAGCGCCTGGCGCT CACCACGGCTCTTTGCGTGTAGGCTGGAGCTGC TTC
dacA KO R	AATTTAATGTAATCAATGATTTTGCCGAAGAAG TTACCTTCCGGGATTTCATGGGAATTAGCCATG GTCC
K-12 *rmlC* KO F	ATGAATGTGATTAGAACTGAAATTGAAGATGTG CTAATTCTGGAGCCAAGGTGTAGGCTGGAGCTG CTTC

K-12 *rmlC* KO R	TCATGCAATTAATTTTAATCTGATAAGCTCATCTAACGTAAAGAGCCTTTATGGGAATTAGCCATGGTCC
wecA KO F	TCGGTTTACGCAGGGATTTGCTTCACGTTCGGAATTGTCGGTGTAGGCTGGAGCTGCTTC
wecA KO R	CTGCGTTTTACGCGCTTAATAAAGCGAGCAACTTTCCAGGATGGGAATTAGCCATGGTCC
waaU KO F	TTAATTAAGAATAAACAAGTTTAAGAAGTGAGTTAAAACATGGGAATTAGCCATGGTCC
waaU KO R	TAATTAATCATCCTGAAACTAAAATAATATGGTATAAAAGTGTAGGCTGGAGCTGCTTC
K-12 *waaL* KO F	TCAACAGTCAAGCAGTTTTGGAAAAGTTATCATCATTATAAAGGTAAAACATGGGAATTAGCCATGGTCC
K-12 *waaL* KO R	TTGTATAGATAAGAAGTGAGTTTTAACTCACTTCTTAAACTTGTTTATTCGTGTAGGCTGGAGCTGCTTC
EC958 *waaL* KO F	TCAACAGTCAAGCAGTTTTGGAAAAGTTATCATCATTATAAAGGTAAAACGTGTAGGCTGGAGCTGCTTC
EC958 *waaL* KO R	ATTAAGTTGTATAGATAAGAAGTGAGTTTTAACTCACTTCTTAAACTTGTATGGGAATTAGCCATGGTCC
CFT073 *waaL* KO F	GTTAGGTCTTGCATTAAATAACCCATCCTCGTAGCATAGGTTGAAATTATGTGTAGGCTGGAGCTGCTTC
CFT073 *waaL* KO R	TAATTTTGAAATAAAATCAGCTTCCTTGCTGATTTTATTTCACATATTCAATGGGAATTAGCCATGGTCC
K-12 *rmlC* cloning F	AATTTCTAGATCATGCAATTAATTTTAATCTGATAAGCTC
K-12 *rmlC* cloning R	AATTGGTACCATGAATGTGATTAGAACTGAAATTGAAG
K-12 *waaL* cloning F	AATTGGTACCATGCTAACATCCTTTAAACTTCATTC
K-12 *waaL* cloning R	AATTGGATCCTTAATTAATTGTATTGTTACGATTATTAATGACG

References to supplementary information

1. McIntosh MA, Chenault SS, Earhart CF. Genetic and physiological studies on the relationship between colicin B resistance and ferrienterochelin uptake in Escherichia coli K-12. J Bacteriol. 1979;137(1):653-7. Epub 1979/01/01. doi: 10.1128/jb.137.1.653-657.1979. PubMed PMID: 153899; PubMed Central PMCID: PMCPMC218495.
2. Qin J, Doyle MT, Tran ENH, Morona R. The virulence domain of Shigella IcsA contains a subregion with specific host cell adhesion function. PloS one. 2020;15(1):e0227425. Epub 2020/01/08. doi: 10.1371/journal.pone.0227425. PubMed PMID: 31910229; PubMed Central PMCID: PMCPMC6946128.
3. Tran EN, Doyle MT, Morona R. LPS unmasking of *Shigella flexneri* reveals preferential localisation of tagged outer membrane protease IcsP to septa and new poles. PloS one. 2013;8(7):e70508. doi: 10.1371/journal.pone.0070508. PubMed PMID: 23936222; PubMed Central PMCID: PMCPMC3723647.
4. Totsika M, Beatson SA, Sarkar S, Phan MD, Petty NK, Bachmann N, et al. Insights into a multidrug resistant Escherichia coli pathogen of the globally disseminated ST131 lineage: genome analysis and virulence mechanisms. PloS one. 2011;6(10):e26578. Epub 2011/11/05. doi: 10.1371/journal.pone.0026578. PubMed PMID: 22053197; PubMed Central PMCID: PMCPMC3203889.
5. Welch RA, Burland V, Plunkett G, 3rd, Redford P, Roesch P, Rasko D, et al. Extensive mosaic structure revealed by the complete genome sequence of uropathogenic Escherichia coli. Proceedings of the National Academy of Sciences of the United States of America. 2002;99(26):17020-4. Epub 2002/12/10. doi: 10.1073/pnas.252529799. PubMed PMID: 12471157; PubMed Central PMCID: PMCPMC139262.
6. Martinez E, Bartolome B, de la Cruz F. pACYC184-derived cloning vectors containing the multiple cloning site and lacZ alpha reporter gene of pUC8/9 and pUC18/19 plasmids. Gene. 1988;68(1):159-62. Epub 1988/08/15. doi: 10.1016/0378-1119(88)90608-7. PubMed PMID: 2851489.
7. Guzman LM, Belin D, Carson MJ, Beckwith J. Tight regulation, modulation, and high-level expression by vectors containing the arabinose PBAD promoter. J Bacteriol. 1995;177(14):4121-30. Epub 1995/07/01. doi: 10.1128/jb.177.14.4121-4130.1995. PubMed PMID: 7608087; PubMed Central PMCID: PMCPMC177145.
8. Datsenko KA, Wanner BL. One-step inactivation of chromosomal genes in Escherichia coli K-12 using PCR products. Proceedings of the National Academy of Sciences of the United States of America. 2000;97(12):6640-5. doi: DOI 10.1073/pnas.120163297. PubMed PMID: WOS:000087526300074.
9. Larue K, Ford RC, Willis LM, Whitfield C. Functional and structural characterization of polysaccharide co-polymerase proteins required for polymer export in ATP-binding cassette transporter-dependent capsule biosynthesis pathways. The Journal of biological chemistry. 2011;286(19):16658-68. Epub 2011/04/02. doi: 10.1074/jbc.M111.228221. PubMed PMID: 21454677; PubMed Central PMCID: PMCPMC3089508.
10. Hong Y, Reeves PR. Diversity of o-antigen repeat unit structures can account for the substantial sequence variation of wzx translocases. J Bacteriol. 2014;196(9):1713-22. Epub 2014/02/18. doi: 10.1128/JB.01323-13. PubMed PMID: 24532778; PubMed Central PMCID:

PMCPMC3993327.
11. Davison J, Heusterspreute M, Chevalier N, Ha-Thi V, Brunel F. Vectors with restriction site banks. V. pJRD215, a wide-host-range cosmid vector with multiple cloning sites. Gene. 1987;51(2-3):275-80. Epub 1987/01/01. doi: 10.1016/0378-1119(87)90316-7. PubMed PMID: 3036654.
12. Morona R, Mavris M, Fallarino A, Manning PA. Characterization of the rfc region of Shigella flexneri. J Bacteriol. 1994;176(3):733-47. Epub 1994/02/01. doi: 10.1128/jb.176.3.733-747.1994. PubMed PMID: 7507920; PubMed Central PMCID: PMCPMC205111.
