# Supplementary material for: O antigen biogenesis sensitises Escherichia coli K-12 to bile salts, providing a plausible explanation for its evolutionary loss
Source: PLoS Genet. 2023 Oct 4;19(10):e1010996. doi: 10.1371/journal.pgen.1010996 (PMC10578602; doi:10.1371/journal.pgen.1010996)
Supplement: S2 Table — (PDF) [file pgen.1010996.s002.pdf]

S2 Table. Differences of MG1655 used in this study and reference MG1655 (U00096)

**Parent MG1655**		
**Mutations**	**locus in U00096**	**Note**
Deletion of Insertion element between *frsA* and *crl*	257927-258623	
Deletion of mobile element between *ychE* and *oppA*	1299498-1300694	
Deletion between *ynaJ* and *dbpA*	1397459-1411026	
Deletion of mobile element between *flhD* and *uspC*	1978516-1979229	
Deletion of 2 bases (CC) in *gatC*	2173363-2173364	Frame shift
Single base (G to T) substitution in *ypjB*	2784096	
Single base (G) insertion in *glpG*	3560455-3560456	Frame shift
Double base insertion (GC) in REP321j region	4296381-4296382	
**Heterogeneous mutations in MG1655-S and Suppressor Mutants**		
**Mutations**	**locus in U00096**	**Note**
Single base (A to T) substitution in *wbbL* (MG1655-S, BP2, BP5, BP11, BP27, BP28, BP30)	2101747	synonymous
Inversion between *ycfK* and *stfE* (BP16, BP27, BP28, BP30,	1207790-1207805	e14 prophage phase variation[1]

References to supplementary information

1. Goldberg A, Fridman O, Ronin I, Balaban NQ. Systematic identification and quantification of phase variation in commensal and pathogenic Escherichia coli. Genome Med. 2014;6(11):112. Epub 2014/12/23. doi: 10.1186/s13073-014-0112-4. PubMed PMID: 25530806; PubMed Central PMCID: PMCPMC4272514.
