# Supplementary figures and images for: O antigen biogenesis sensitises Escherichia coli K-12 to bile salts, providing a plausible explanation for its evolutionary loss
Source: PLoS Genet. 2023 Oct 4;19(10):e1010996. doi: 10.1371/journal.pgen.1010996 (PMC10578602; doi:10.1371/journal.pgen.1010996)

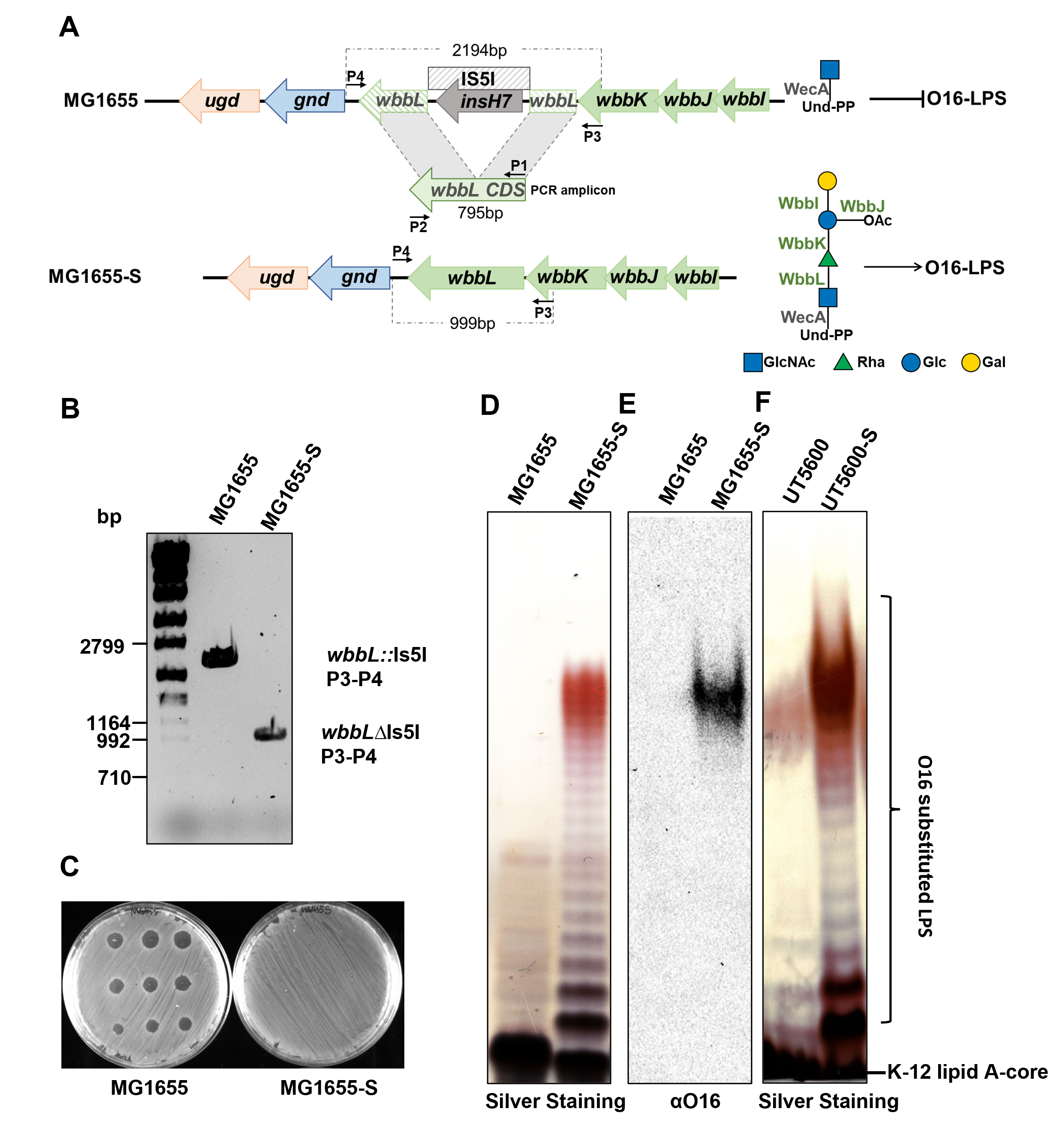

Supplement: S1 Fig — (A) Schematic representation of the strategy employed for wbbL allelic replacement in the rfb gene cluster in MG1655 to construct strain MG1655-S. The respective O16 repeating units that would be produced in the cytosol denote the expected LPS products by each strain. Primers P1 and P2 used to generate MG1655-S are mapped onto rfb regions by black arrows. (B) PCR amplicons of wbbL region from MG1655 and MG1655-S (using primers P3 and P4) confirming the replacement of an intact wbbL CDS in MG1655-S. (C) Colicin sensitivity assay confirming increased resistance of MG1655-S due to regained O antigen substituted LPS production. Colicin E2 was used at 1 mg/ml and in subsequent 2-fold dilutions (5 μl spots). (D&F) Silver stained SDS-PAGE of LPS samples from two sets of E. coli K-12 strains (wild-type MG1655 and UT5600 carry the IS51 element in wbbL and MG1655-S/UT5600-S are isogenic wbbL intact strains, respectively). LPS patterns confirm restored production of O antigen substituted LPS in the engineered MG1655-S and UT5600-S strains. (E) Western immunoblotting of samples as in (D) with anti-O16 antibodies showing the restoration of O16 O antigen production in MG1655-S. (TIF) [file pgen.1010996.s003.tif]

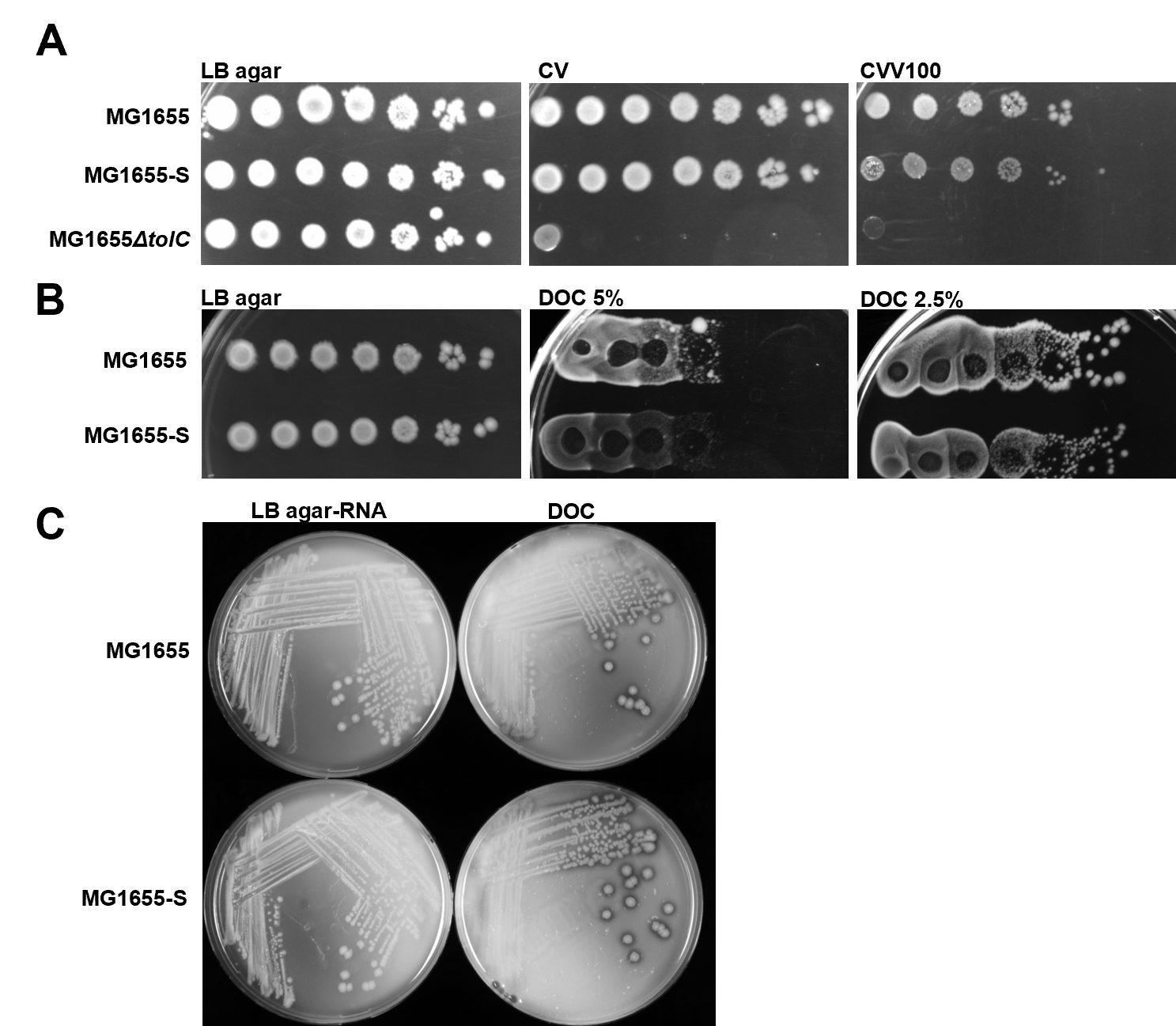

Supplement: S2 Fig — (A&B) Bacterial cultures of indicated strains grown in LB media were adjusted to OD600 of 1 and spotted (4 μl) in 10-fold serial dilutions (100 to 10−6) onto LB agar supplemented with 0.0001% (w/v) crystal violet (CV) or 100 μg/ml vancomycin and CV (CVV100), or 5% and 2.5% (w/v) sodium deoxycholate (DOC). (C) RNase I leakage assay of MG1655 and MG1655-S grown on LB agar supplemented without or with 0.1% (w/v) DOC. (TIF) [file pgen.1010996.s004.tif]

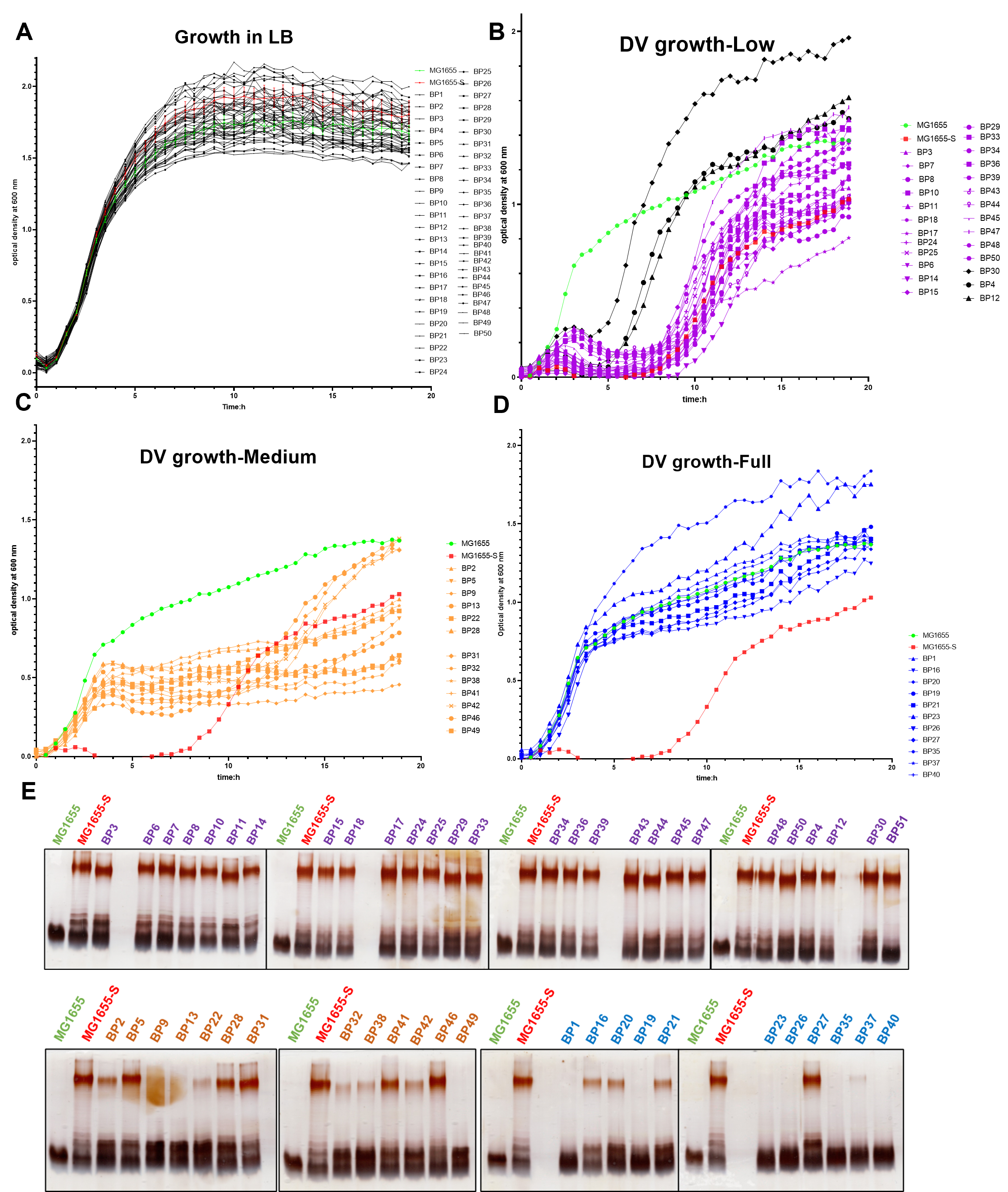

Supplement: S3 Fig — Growth curve of MG1655, MG1655-S and BP1-BP51 suppressor mutants in LB media (A) or in LB supplemented with (B-D) 100 μg/ml vancomycin and 0.1% (w/v) DOC (DV100). E) Silver staining of SDS-PAGE of LPS samples prepared from MG1655, MG1655-S and BP1-BP51 suppressor mutants. (TIF) [file pgen.1010996.s005.tif]

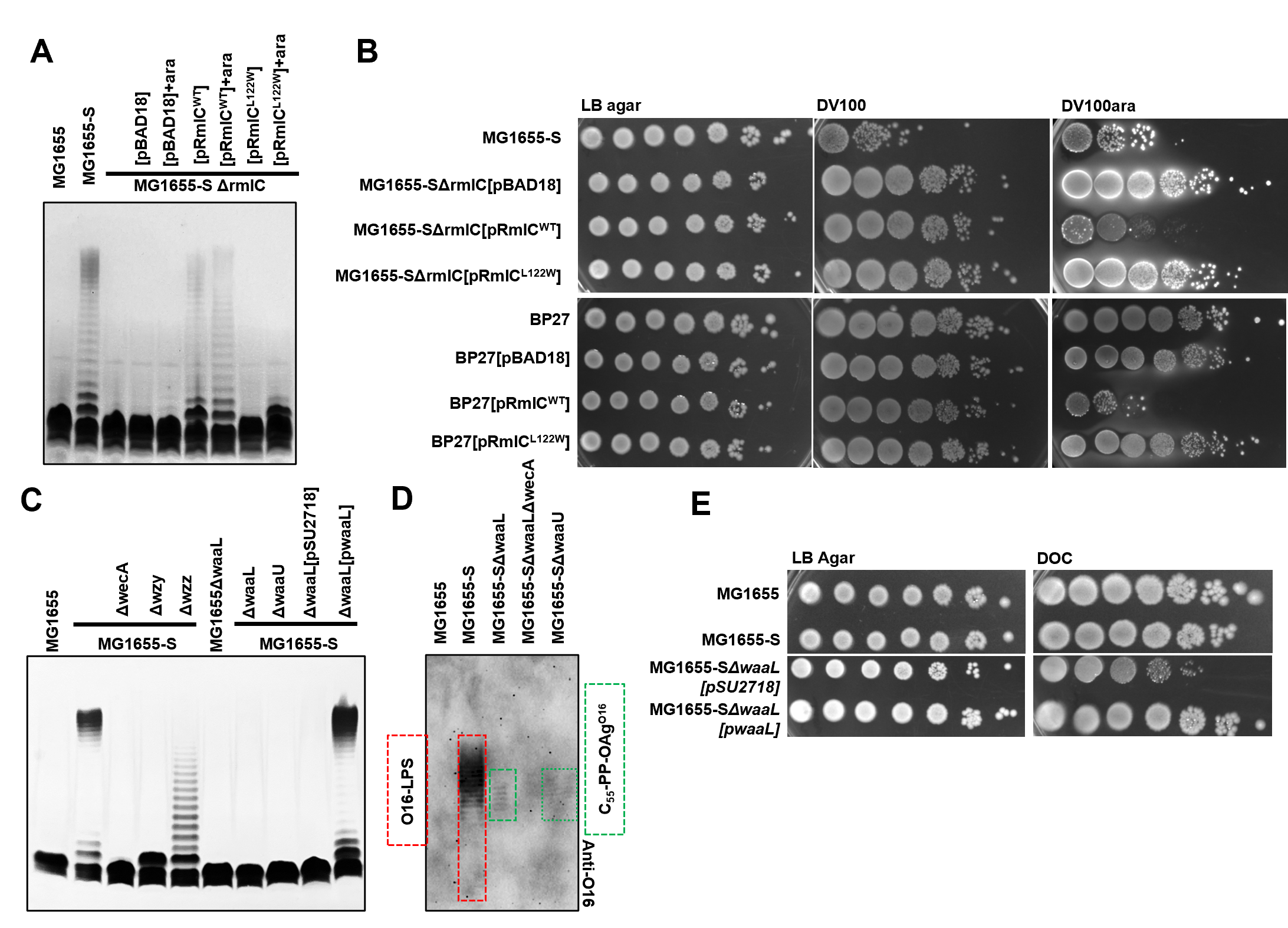

Supplement: S4 Fig — (A & C) Silver staining of SDS-PAGE of LPS samples of MG1655, MG1655-S and their mutational derivatives. Supplementation of media with 0.2% (w/v) arabinose was used in samples as indicated (+ara). Bacterial cultures of indicated strains (B & E) grown in LB media were adjusted to OD600 of 1 and spotted (4 μl) in 10-fold serial dilutions (100 to 10−6) onto LB agar supplemented without or with 0.1% (w/v) DOC and 100 μg/ml vancomycin (DV100) in the absence or presence of 0.2% (w/v) L-arabinose (DV100ara). (D) Western immunoblotting of proteinase K-treated LPS samples with anti-O16 antibodies showing OAgO16-capped LPS in MG1655-S (red dashed box) and the accumulated C55-PP-OAgO16 intermediates (green dashed box) in MG1655-S ΔwaaL and ΔwaaU mutants. (TIF) [file pgen.1010996.s006.tif]
